# Tailoring Dense, Orientation–Tunable, and Interleavedly Structured Carbon‐Based Heat Dissipation Plates

**DOI:** 10.1002/advs.202205962

**Published:** 2023-01-10

**Authors:** Lianqiang Peng, Huitao Yu, Can Chen, Qingxia He, Heng Zhang, Fulai Zhao, Mengmeng Qin, Yiyu Feng, Wei Feng

**Affiliations:** ^1^ School of Materials Science and Engineering Tianjin Key Laboratory of Composite and Functional Materials Tianjin University Tianjin 300350 P. R. China; ^2^ Key Laboratory of Materials Processing and Mold Ministry of Education Zhengzhou University Zhengzhou 450002 P. R. China

**Keywords:** carbon nanotube arrays, graphene films, hierarchical building blocks, interfacial fusion, interleaved structure

## Abstract

The controllability of the microstructure of a compressed hierarchical building block is essential for optimizing a variety of performance parameters, such as thermal management. However, owing to the strong orientation effect during compression molding, optimizing the alignment of materials perpendicular to the direction of pressure is challenging. Herein, to illustrate the effect of the ordered microstructure on heat dissipation, thermally conductive carbon‐based materials are fabricated by tailoring dense, orientation–tunable, and interleaved structures. Vertically aligned carbon nanotube arrays (VACNTs) interconnected with graphene films (GF) are prepared as a 3D core‐ordered material to fabricate compressed building blocks of O–VA–GF and S–VA–GF. Leveraging the densified interleaved structure offered by VACNTs, the hierarchical O–VA–GF achieves excellent through‐plane (41.7 W m^−1^ K^−1^) and in‐plane (397.9 W m^−1^ K^−1^) thermal conductivities, outperforming similar composites of S–VA–GF (through‐plane: 10.3 W m^−1^ K^−1^ and in‐plane: 240.9 W m^−1^ K^−1^) with horizontally collapsed carbon nanotubes. As heat dissipation plates, these orderly assembled composites yield a 144% and 44% enhancement in the cooling coefficient compared with conventional Si_3_N_4_ for cooling high‐power light‐emitting diode chips.

## Graphical abstract

Based on the force‐thermal coupling design concept by using covalently interconnected carbon nanotubes–graphene, the fabricated hierarchical compressed building blocks (VA–GF) exhibit high thermal conductivity and excellent mechanical properties. As heat dissipation plates for cooling high‐power light‐emitting diode chips, the thermal management performance of the VA–GF significantly exceeds that of conventional Si_3_N_4_.

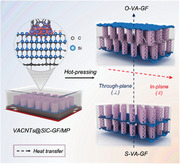

## 1 Introduction

The dramatic temperature rise induced by ultra‐high heat‐flow density has manifested as a bottleneck problem limiting the development of high‐power, highly integrated devices, particularly because the heat flow of the devices tends to be periodic, non‐uniform, and significantly different in direction.^[^ ^1^ ^]^ The design of highly thermally conductive heat dissipation materials is critical to ensure the operational efficiency, reliability, and longevity of the devices.^[^ ^2^ ^]^ In several candidate materials, hierarchical and structurally controllable carbon‐based building blocks are considered a valuable framework for designing advanced thermal management materials and their integrated devices.^[^ ^3^ ^]^ Owing to the structure‐dependent heat conduction characteristics of low‐dimensional carbon materials, where phonons can solely be efficiently transferred in a single direction along their lattice, modulating the orientation and distribution of materials can optimize the heat dissipation capability and orientation of hierarchical building blocks.^[^ ^3^, ^4^ ^]^ However, the pressure effect during the molding process typically affects the material orientation and distribution, thereby causing the compressed building blocks to exhibit heat‐transfer capabilities with different dimensions.^[^ ^5^ ^]^ Therefore, modulating the micro‐nano structure during compression molding is a crucial strategy for optimizing the multidimensional heat‐transfer capability of hierarchical compressed building blocks.

The heat conduction of isotropic materials is uniform in all directions, contributing to the formation of a homogeneous temperature distribution.^[^ ^6^ ^]^ For example, employing the commonly used technique of applying isostatic pressure for preparing carbon‐based compressed building blocks, the materials can form isotropic structures subjected to equal pressure in all directions transmitted by the fluid medium during the assembly process, thereby granting these compressed building blocks with a multidimensional and uniform heat‐transfer capability. However, materials are generally oriented randomly during the isostatic compression process, forming an uncontrollable hierarchical structure and reducing the orientation tunability, which hinders the construction of effective heat‐transfer pathways. The thermal conductivity of isostatically compressed carbon‐based building blocks is typically less than 200 W m^−1^ K^−1^, which limits their directional heat‐transfer capability.^[^ ^7^ ^]^ Moreover, the fabrication of hierarchical compression members featuring high thermal conductivity, using the strategy of directional compression, has proven to be very effective. In particular, for compressed building blocks assembled via directional hot‐pressing of expanded graphite intercalated with carbon nanotubes (CNTs), the in‐plane thermal conductivity exceeds 300 W m^−1^ K^−1^,^[^ ^8^ ^]^ thereby contributing to efficient phonon transfer. However, during directional compression molding, the weaker interaction between the materials results in structural instability, which in turn results in a tendency to orient perpendicular to the pressure direction.^[^ ^9^ ^]^ Moreover, the through‐plane thermal conductivity of such compressed building blocks is considerably lower than that in the desired direction, thereby yielding highly anisotropic heat‐transfer characteristics.^[^ ^10^ ^]^ The improvement in the unidirectional heat‐transfer performance is typically concurrent with the decrement of the heat‐transfer performance in non‐preferred directions,^[^ ^11^ ^]^ which remains a great contradiction that building blocks fabricated by the directional compression strategy generally yield a non‐uniform and low heat flux heat dissipation process as a result of applying high‐power heat sources.^[^ ^12^ ^]^

Interconnected multi‐directional phonon pathways will be produced by optimizing the orientation and interfacial contact of the multi‐level structure and controlling the stability of the micro‐nano structure during compression molding. This is anticipated to result in superior, uniform, and multidimensional thermal conductivity while weakening the strong orientation effect of pressure.^[^ ^13^ ^]^ Therefore, improving the interaction or support between the materials is an effective measure for enhancing the stability of the micro‐nano structures during the compression molding process.^[^ ^14^ ^]^ Kim et al. showed that the multi‐level heat‐transfer pathways within the as‐prepared compressed building blocks were considerably optimized by coating the surface of polyphenylene sulfide particles with epoxy resin and subsequently adsorbing hexagonal boron nitride nanosheets to pre‐construct the core‐shell support structure.^[^ ^15^ ^]^ Lv et al. used the carbon nanotubes–expanded graphite coated with graphene oxide to construct a micro–nano structure followed by hot–pressing, which enhanced the through‐plane thermal conductivity of the composites.^[^ ^16^ ^]^ Therefore, constructing an ideal support structure with strong interactions is essential for optimizing the multidimensional heat‐transfer capability of hierarchical compressed building blocks.^[^ ^17^ ^]^ However, the above micro‐nano structures have no direction‐selectivity in the subsequent compression assembly process, resulting in disordered stacking. Therefore, it is still challenging to effectively regulate the microscopic orientation within the materials during the compression molding, restricting the templated customizability of compressed hierarchical building blocks to some extent.

In this study, based on in situ chemical vapor deposition and annealing strategies, we synthesized an ordered and thermally conductive 3D carbon framework (VACNTs@SiC–GF) comprising VACNTs–graphene film (GF) interconnected via SiC. The symmetrical and multi‐directionally aligned 3D framework (i.e., horizontally and vertically) can maximally utilize the anisotropic thermal conductivity of graphene and CNTs, yielding macroscopically uniform and orthogonal phonon pathways but microscopically straightforward. Subsequently, the VACNTs@SiC framework was impregnated with mesophase pitch (MP) to form stable VACNTs@SiC–GF/MP. By regulating the assembly method of VACNTs@SiC–GF/MP based on the design concept of force–thermal coupling, two novel carbon‐based composites with high density, tunable thermal conductivity, and excellent mechanical strength were prepared via a hot‐pressing process with the characteristics of VACNTs aligned to VACNTs (O–VA–GF) and VACNTs aligned to GF (S–VA–GF). The resulting block of O–VA–GF exhibits a hierarchical structure in which the internal interlocking CNTs form a dense fused interface. This results in the generation of more continuous heat‐transfer pathways, with the horizontal GF at the top and bottom sides increasing the contact area with the mating surfaces to enhance the heat flux. Moreover, the intense interfacial interactions further contributed to the excellent tensile and bending strengths of the O–VA–GF. The hierarchical structure of S–VA–GF consisting of horizontally collapsed CNTs produced a carbon nanotube–graphene heterogeneous interface featuring weak interfacial interactions, thereby exhibiting weaker interfacial phonon mobility and lower mechanical strength compared with O–VA–GF. In the heat dissipation performance test, the cooling efficiency of the systems integrated with O–VA–GF or S–VA–GF was significantly higher than that of conventional Si_3_N_4_. The results obtained in this study validate that our proposed composites show great potential as carbon‐based heat dissipation plates for high‐flux heat dissipation applications in high‐power devices.

## 2 Results and Discussion

### 2.1 Synthesis and Structural Characterization of VACNTs@SiC–GF

**Figure 1:**
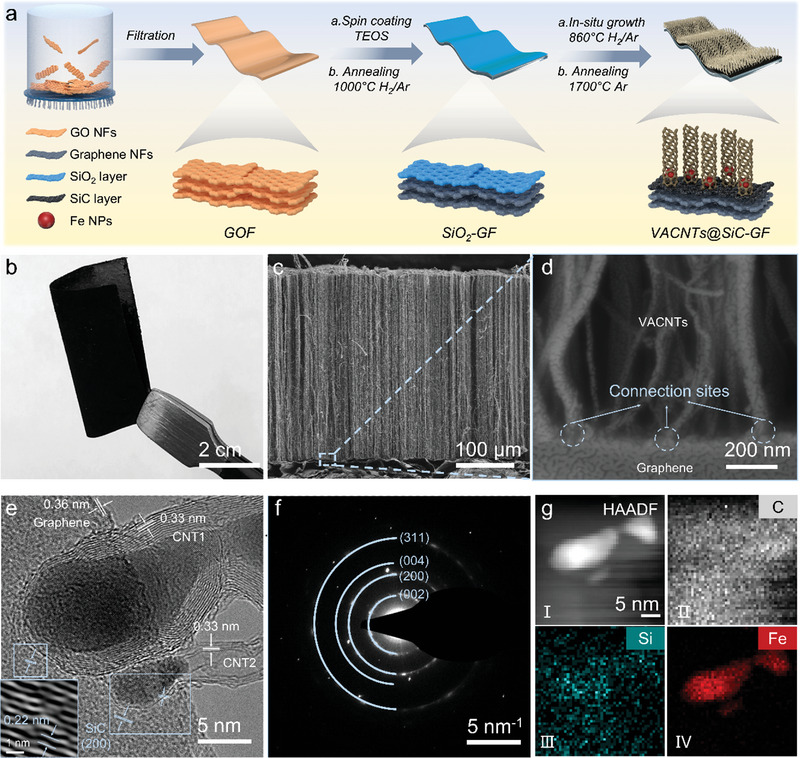
a) Schematic of the fabrication process and b) optical photograph, and c) the cross‐sectional scanning electron microscopy (SEM) image of VACNTs@SiC–GF. d) High‐resolution SEM image, e) HRTEM image, f) SAED pattern, and g) energy‐dispersive spectrometry (EDS) mapping of the connection sites of graphene and CNTs.

**Figure** **1**a presents the strategy for developing the 3D interconnected structure of VACNTs@SiC–GF and the corresponding structural changes for each step of the fabrication process. In brief, a graphene oxide film (GOF) is prepared with high‐quality monolayer graphene oxide nanosheets via simple vacuum filtration, demonstrating excellent flexural flexibility and a dense cross‐sectional structure featuring a layer‐wise development (Figure S1, Supporting Information). After pyrolysis of tetraethyl orthosilicate (TEOS) spin‐coated onto the GOF surface, the GOF is converted to matt silver, while a dense SiO_2_ layer is uniformly formed on the surface of GF (SiO_2_–GF), as presented in Figure S2 (Supporting Information). Furthermore, 3D VACNTs@SiO_2_–GF is fabricated via the growth of VACNTs onto the surface of SiO_2_–GF. Following annealing based on a carbothermal reduction reaction,^[^ ^18^ ^]^ a SiC layer between the GF and VACNTs is created to form interconnected VACNTs@SiC–GF. The chemical mechanism of the SiO_2_ to SiC conversion at the VACNTs–GF interface can be explained according to the flowing reactions:^[^ ^19^ ^]^

(1) $${\mathrm{SiO}}_{2\left(s\right)}+3{\;C}_{\left(s\right)}={\mathrm{SiC}}_{\left(s\right)}+2{\mathrm{CO}}_{\left(g\right)}$$

It is generally accepted that SiC is formed through the intermediate product of SiO. Therefore, the above reaction can also be decomposed into two basic reaction steps:

(2) $${\mathrm{SiO}}_{2\left(s\right)}+C_{\left(s\right)}={\mathrm{SiO}}_{\left(g\right)}+{\mathrm{CO}}_{\left(g\right)}$$

(3) $${\mathrm{SiO}}_{\left(g\right)}+2C_{\left(s\right)}={\mathrm{SiC}}_{\left(s\right)}+{\mathrm{CO}}_{\left(g\right)}$$

Furthermore, the morphology of VACNTs@SiC–GF is characterized in Figure 1b–g. As illustrated in Figure 1b, after the VACNTs growth and annealing, the VACNTs@SiC–GF turns black in color and remains extremely bendable and flexible. The morphologies of the VACNTs@SiC–GF are presented in Figure 1c,d, and Figure S3 (Supporting Information). Observably, the CNTs form perfectly vertical arrays, the bottom of which form stable connection sites with GF. Moreover, high‐resolution transmission electron microscopy (HRTEM) further reveals a stable connection pattern between the VACNTs and GF. Before observation, the VACNTs@SiC–GF heterojunctions are dispersed in ethanol and ultrasonicated for 30 min. Figure 1e shows that the CNTs remain tightly anchored to graphene. The clear crystal features of SiC, CNTs, and graphene are evident at the connection sites with d‐spacing values of 0.22, 0.33, and 0.36 nm corresponding to (200), (002), and (002), respectively (Figure S4, Supporting Information). Moreover, the diffraction rings in the selected area electron diffraction (SAED) pattern can be indexed to the reflections of the aforementioned structure (Figure 1e,f). Figure 1g shows the Energy‐dispersive X‐ray spectroscopy analysis results of the surface elements (C, Si, and Fe) of the connection sites, which further establishes the aforementioned connection. SiC is distributed uniformly at the interface between the graphene and CNTs. And the Fe element is introduced by the high‐temperature pyrolysis of the ferrocene precursor for the catalytic growth of CNTs.

**Figure 2:**
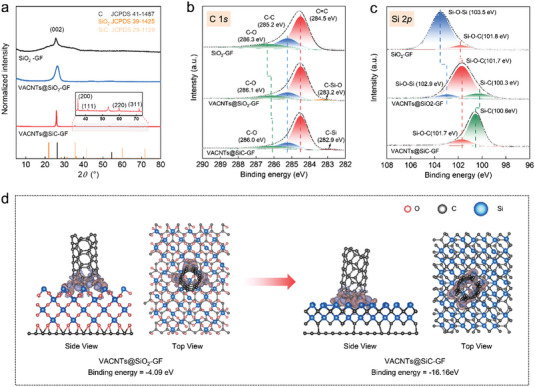
a) XRD patterns, b) high‐resolution C 1*s* spectra, and c) high–resolution Si 2*p* spectra of various samples. d) Calculated charge density of VACNTs@SiO_2_–GF and VACNTs@SiC–GF. The isosurface contours correspond to 0.005 e Å^−3^.

To comprehensively investigate the formation of the connections between the VACNTs and GF, the crystal information and chemical composition of the derived heterogeneous interfaces were characterized. In **Figure** **2**a, except for the signal at 26.5° originating from the (002) plane of the graphene nanosheets, the crystalline features corresponding to SiO_2_ notably become less ambiguous or even disappear entirely after annealing. Simultaneously, a series of diffraction peaks at 36°, 42°, 61°, and 72° are assigned to the (111), (200), (220), and (311) planes of 3C‐SiC, respectively. This trend indicates that annealing effectively induced the successful conversion of SiO_2_ on the surface of GF to 3C‐SiC.^[^ ^20^ ^]^

The elemental changes and chemical bonding transitions of the materials were characterized using X‐ray photoelectron spectroscopy (XPS). Figure S5 (Supporting Information) depicts the C 1*s*, O 1*s*, Si 2*p*, and Si 2*s* characteristic signals of the materials. The high‐resolution C 1*s* and Si 2*p* spectra are deconvoluted into the main components, as depicted in Figure 2b,c and Table S1 (Supporting Information). The appearance of the peak at 101.8 eV indicates that the SiO_2_, obtained from pyrolysis of TEOS, is associated with the GF to form a Si–O–C covalent connection at the interface.^[^ ^21^ ^]^ After the growth of CNTs, the binding energy of the C–O (286.1 eV) peak of VACNTs@SiO_2_–GF is lower than that of SiO_2_–GF (286.3 eV). And the binding energy of Si–O–Si and Si–O–C peaks is reduced by 0.6 and 0.1 eV, respectively, compared with that of SiO_2_–GF. Moreover, the signals of the C–Si–O and Si–C peaks appear at 283.2 and 100.3 eV, respectively.^[^ ^22^ ^]^ Therefore, the Si–C covalent bonding generated by CNTs with the surface SiO_2_ weakens the bonding strength of Si–O–Si and Si–O–C.^[^ ^23^ ^]^ Notably, as indicated in Figure 2c, the signal of the Si–O–Si peak disappears in the VACNTs@SiC–GF obtained after annealing, whereas the binding energy of the Si–C peak is significantly higher than that before annealing. This trend is related to the appearance of the 3C‐SiC crystalline phase, as revealed through X‐ray diffraction (XRD) analysis. Furthermore, relatively strong covalent linkages can be achieved at the interfaces between graphene–SiC and CNTs–SiC. Thus, the combination is mainly attributed to the SiO_2_–to–SiC conversion at the VACNT–GF interface, leading to the formation of covalent C–Si or C–C bonds, thereby yielding a covalent connection between the VACNTs and GF.^[^ ^24^ ^]^

The results of density functional theory‐based calculations are depicted in Figure 2d and Figure S6 (details are shown in Figures S6–S8 and Table S2, Supporting Information). Observably, compared with the heterogeneous interface of VACNTs–GF connected by SiO_2_ with a binding energy of ‐4.09 eV, the formation of SiC in the VACNTs@SiC–GF has a higher binding energy of ‐16.16 eV. The results indicate that an asymmetric charge distribution results in stronger interfacial interactions at the interface between the CNTs and SiC, thereby intensifying the polarization.^[^ ^25^ ^]^ Strong interfacial interactions contribute to reducing the phonon scattering effect at the interface and improve the interfacial heat‐transfer performance of the heterostructure.^[^ ^26^ ^]^ In Figure S9 (Supporting Information), compared with the thermal diffusivity (22.3 mm^2^ s^−1^) and thermal conductivity (17.0 W m^−1^ K^−1^) of VACNTs@SiO_2_–GF, the thermal diffusivity (52.6 mm^2^ s^−1^) and thermal conductivity (32.4 W m^−1^ K^−1^) of VACNTs@SiC–GF are increased by 136% and 91%, respectively. Therefore, the construction of the highly thermally conductive covalently bonded interface is of significance for the phonon transport across graphene and CNTs.^[^ ^27^ ^]^ In summary, we propose that the construction of covalent interfacial SiC heterojunctions between VACNTs and GF is the preferred 3D ordered thermally conductive carbon‐based framework, which plays a crucial role in enhancing the interaction between materials, thereby improving the stability of the thermally conductive framework, while constructing 3D continuous and orthogonal phonon pathways to enhance the multidimensional heat‐transfer performance.

### 2.2 Fabrication and Thermal Properties of VA–GF Composites

**Figure 3:**
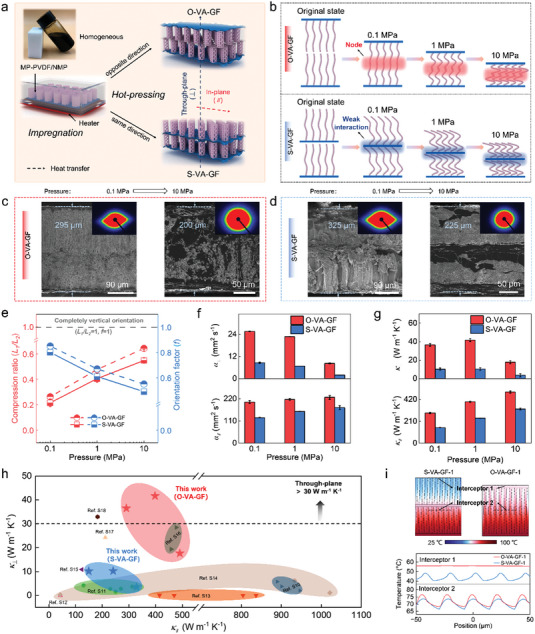
a) Schematic of the fabrication process, b) and the deformation mechanisms of the O–VA–GF and S–VA–GF under different pressure conditions (0.1, 1, and 10 MPa). The typical SEM images and SAXS patterns of c) O–VA–GF, and d) S–VA–GF with pressures of 0.1 and 10 MPa, respectively. e) The compression ratio and the orientation factor of the internal VACNTs of O–VA–GF and S–VA–GF as a function of pressure. f) *α*_∥_ and *α*_⊥_, as well as g) *κ*_∥_ and *κ*_⊥_of the O–VA–GF and S–VA–GF as a function of pressure. h) Comparison of *κ*_∥_ and *κ*_⊥_ of the O–VA–GF and S–VA–GF with the reported carbon‐based HDMs. i) The simulated transient‐temperature distribution of O–VA–GF–1 and S–VA–GF–1.

VACNTs@SiC–GF is considered a core–ordered thermally conductive framework for improving the thermal conductivity of materials. Herein, two hierarchical carbon‐based materials are developed by regulating the compound process to illustrate the effect of ordered microstructure on heat dissipation. The VACNTs@SiC–GF framework (thickness of ≈210 µm) is compounded with a homogeneous dispersion of MP–PVDF/NMP via thermal impregnation in a Teflon mold. The optimal wettability between the dispersion and the framework ensures that the MP is successfully incorporated into the gaps of the VACNTs (Figure S10, Supporting Information). Subsequently, a pair of frameworks are assembled with a partially ordered structure in different directions via hot‐pressing to fabricate compressed building blocks of O–VA–GF and S–VA–GF (**Figure** **3**a).

In addition, we investigate the effect of the arrangement of VACNTs during compression molding on the heat‐transfer performance of the materials. A series of O–VA–GF (O–VA–GF–0.1, O–VA–GF–1, and O–VA–GF–10) and S–VA–GF (S–VA–GF–0.1, S–VA–GF–1, and S–VA–GF–10) are prepared by regulating the pressure (0.1, 1, and 10 MPa) and changing the compression ratio (*L*_1_/*L*_2_) during the hot‐pressing process. The entire gradient‐compression process can be understood on the microscopic scale in combination with the structural and material characteristics of VACNTs@SiC–GF (Figure 3b). Without directional compression (original state), the CNTs are vertically oriented. Moreover, the CNTs within O–VA–GF and S–VA–GF undergo different orientation processes under compression, leading to structural evolution. Notably, the VACNTs at the O–VA–GF interface can be embedded to form a homogeneous fusion interface. The contacting CNTs might form supporting nodes during insertion, which can relieve the deformation caused by pressure to ensure that the CNTs are oriented along the vertical direction.^[^ ^14^, ^28^ ^]^ For S–VA–GF, both the inner CNTs and top CNTs are strongly oriented horizontally by the stress compression of the large contact area. Moreover, a weakly interacting VACNTs–GF heterogeneous interface is formed internally. Following the gradient–compression molding, both the O–VA–GF and S–VA–GF form dense, orientation–tunable, and interleaved hierarchical structures featuring densities greater than 1 g cm^−3^ (O–VA–GF–0.1: 1.32 g cm^−3^, O–VA–GF–1: 1.57 g cm^−3^, O–VA–GF–10: 1.73 g cm^−3^; S–VA–GF–0.1: 1.21 g cm^−3^, S–VA–GF–1: 1.46 g cm^−3^, S–VA–GF–10: 1.58 g cm^−3^), as shown in Figure S11 (Supporting Information).

The formation of the different dense hierarchical structures, as mentioned previously, was characterized using scanning electron microscopy (SEM), and the orientation process of the CNTs was investigated via small‐angle X‐ray scattering (SAXS) (Figure 3c,d, and Figure S11, Supporting Information). When the pressure is increased from 0.1 MPa to 10 MPa, a weaker vertical orientation behavior of the arrayed CNTs in SEM, and a weaker anisotropy of the SAXS patterns are observed.^[^ ^29^ ^]^ This result indicates that the CNTs within the O–VA–GF and S–VA–GF are gradually horizontally oriented. However, the vertical orientation and structural integrity of the CNTs within O–VA–GF are consistently superior to those of S–VA–GF under the same pressure conditions. The compression ratio of the O–VA–GF is higher than that of the S–VA–GF (Figure 3e), which can be explained by the insertion of CNTs and the formation of nodes, as illustrated in Figure 3b. This compelling process of structural evolution is also evidenced by calculating Hermans orientation factor (*f*) for the analysis of the alignment of the material obtained from the azimuthal angle distributions (Figure S13, Supporting Information).^[^ ^29^, ^30^ ^]^ An *f*‐value of 1 indicates a completely vertical orientation of the CNTs. With an increase in pressure, the *f*‐value of both complexes gradually decreases, but the *f*‐value of O–VA–GF is greater than that of S–VA–GF. This trend suggests that the arrayed CNTs within the former can maintain a better vertical orientation under the strong orientation effect of pressure. Therefore, O–VA–GF and S–VA–GF with different hierarchical structures of dense, orientation–tunable, and interleaved structures can be tailored to optimize heat dissipation performance by regulating the pressure‐induced molding process.

To investigate the correlation between the microstructure and thermal conductivity of the compressed building blocks, the thermal diffusivities (*α*) of the O–VA–GF and S–VA–GF are measured using the laser flash technique. As demonstrated in Figure 3f, the in‐plane thermal diffusivity (*α*_∥_) of O–VA–GF and S–VA–GF increases with pressure. However, the through‐plane thermal diffusivity (*α*_⊥_) decreases with increasing pressure. This trend is attributed to the horizontal orientation of the CNTs during the stress‐induced orientation process. Moreover, the hierarchical structure of O–VA–GF is composed of internally interleaved CNTs forming a fusion interface, yielding more continuous heat‐transfer pathways, thereby allowing phonons at the interface to exhibit fast vibrations along the vertical direction and reducing scattering in the horizontal direction. For S–VA–GF, both the inner CNTs and top CNTs are strongly horizontally oriented by the stress compression of the large contact area. Moreover, its internal VACNTs–GF heterogeneous interface interacts weakly, which hinders the interface slip for effectively transferring phonons. This result indicates that *α*_∥_ and *α*_⊥_ of O–VA–GF are higher than those of S–VA–GF at the same level of pressure. For comparison, the corresponding thermal conductivities (*κ*) of the materials are calculated using the following equation:

(4) $$\kappa =\alpha \times \rho \times C_{p}$$

where *ρ* denotes the density and *C*_p_ represents the specific heat capacity determined by the DSC–based sapphire method (Figure S14, Supporting Information).^[^ ^31^ ^]^ Figure 3g plots the in‐plane thermal conductivity (*κ*_∥_) and through‐plane thermal conductivity (*κ*_⊥_) of O–VA–GF and S–VA–GF as a function of pressure. The *κ*_∥_ of both increases with increasing pressure because of the increasing *ρ*, *C*_p_ and *α*_∥_. However, as the pressure increases from 0.1 MPa to 1 MPa, the increased percentages of the *C*_P_ × *ρ* value of O–VA–GF–1 (32.4%) and S–VA–GF–1 (33.0%) are higher than the decreased percentages of the *α*_⊥_ of O–VA–GF–1 (12.7%) and S–VA–GF–1 (21.4%). Thus, the trend of *κ*_⊥_ is increasing when the pressure increases from 0.1 MPa to 1 MPa. However, as the pressure increases from 1 MPa to 10 MPa, the increased percentages of the *C*_P_ × *ρ* value of O–VA–GF–10 (17.7%) and S–VA–GF–1 (18.2%) are much lower than the decreased percentages of the *α*_⊥_ of O–VA–GF–10 (63.8%) and S–VA–GF–10 (71.5%), resulting in a significant reduction in their *κ*_⊥_. Therefore, the *κ*_⊥_ of O–VA–GF and S–VA–GF increases and then decreases with the increasing pressure. Consequently, based on the structural differences generated during compression molding, O–VA–GF–10 and S–VA–GF–10 exhibit the maximal *κ*_∥_ (490.9 and 328.2 W m^−1^ K^−1^, respectively); while, O–VA–GF–1 and S–VA–GF–1 exhibit the maximal *κ*_⊥_ (41.7 and 10.3 W m^−1^ K^−1^, respectively). Compared with previously reported carbon‐based heat dissipation materials (HDMs) (Figure 3h and Table S3, Supporting Information), the composites exhibit excellent *κ*_⊥_ at a relatively high level of *κ*_∥_. In addition, the anisotropic ratio (defined as *κ*_∥_ to *κ*_⊥_) is calculated based on the corresponding *κ*_∥_ and *κ*_⊥_ values to evaluate the thermal transport selectivity of the materials along the in‐plane and through‐plane directions. As indicated in Table S3 (Supporting Information), the anisotropic ratio of O–VA–GF is lower than that of S–VA–GF, which demonstrates that the interleaved structure is significantly advantageous for overcoming the strongly uncontrolled anisotropic thermal conductivity generated via stress. This result is particularly beneficial for the thermal management of spot‐like and large‐area heat sources.^[^ ^32^ ^]^ To simplify the comparison of the effects of the differences between the two structures on the thermal management performance, O–VA–GF–1 and S–VA–GF–1 with the highest *κ*_⊥_ are selected as controls for the study. Furthermore, the thermal conductivities of O–VA–GF–1 and S–VA–GF–1 at different temperatures are investigated as shown in Figure S15 (Supporting Information). The *C*_P_ of O–VA–GF–1 and S–VA–GF–1 increases with increasing temperature (‐30—150 °C) (Table S4, Supporting Information). However, in Figure S15a,b (Supporting Information) the trend of *α*_∥_ and *α*_⊥_ of O–VA–GF–1 and S–VA–GF–1 is significantly decreasing, resulting in a decrease in *κ*_∥_ and *κ*_⊥_ with increasing temperature (Figure S15c,d, Supporting Information). Such behavior can be attributed to the increased relaxation time of phonons with increasing temperature and is consistent with the phonon Umklapp scattering mechanism.^[^ ^33^ ^]^ And the *κ*_∥_ and *κ*_⊥_ of O–VA–GF–1 and S–VA–GF–1 are tested at ‐30 °C and 150 °C for 300 min at a 10 min interval, correspondingly. The result indicates that O–VA–GF–1 and S–VA–GF–1 exhibit sufficient reliability and stability of heat conduction at both ‐30 °C and 150 °C, as shown in Figure S15e,f (Supporting Information).

In addition, to further validate that the heat‐transfer efficiency of the O–VA–GF is higher than that of the S–VA–GF, the transient thermal response of the two structures is simulated through finite element analysis (FEA) (the detailed parameter setting and calculation process of this simulation are presented in Figure S16, Supporting Information). When the transient heat‐transfer time is 0.2 ms, the temperature distributions at the same height of the model and at the top are monitored simultaneously. At the same simulation time as that of S–VA–GF–1, the interior of O–VA–GF–1 possesses a higher upper temperature with a uniform temperature distribution as illustrated in Figure 3i and Figure S16 (Supporting Information). This result indicates that the nodes generated by the interleaved structure within O–VA–GF–1 can effectively enhance heat‐transfer efficiency. In particular, the temperature distribution at the top of the former is fairly uniform because the presence of large‐area GF allows heat to spread evenly across the surface. Therefore, tailoring the dense, orientation–tunable, and interleaved structures of hierarchical carbon‐based materials is an essential strategy for optimizing high‐flux heat dissipation.^[^ ^34^ ^]^

**Figure 4:**
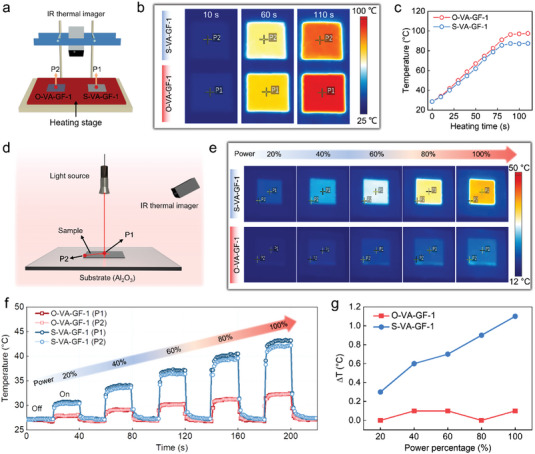
a) Test system configuration for demonstrating transient heat‐transfer performance along the through‐plane direction. b) IR thermal images, and c) corresponding temperature evolution with respect to the heating time of O–VA–GF–1 and S–VA–GF–1. d) Test system configuration for demonstrating transient heat‐transfer performance along the in‐plane direction. e) IR thermal images of steady state, f) corresponding temperature evolution of P1 and P2 with respect to time, as well as g) ΔT at gradient power of O–VA–GF–1 and S–VA–GF–1.

Furthermore, to visually compare the transient heat‐transfer process of the O–VA–GF–1 and S–VA–GF–1 along the through‐plane direction, a pair of compressed building blocks cut to a size of 10 × 10 mm are first placed on a heating stage with an initial temperature of room temperature and then simultaneously heated to 100 °C (**Figure** **4**a). The evolution of the surface temperature with the heating time is recorded using an infrared (IR) thermal imager (Figure 4b,c). Observably, the surface temperature of O–VA–GF–1 increases faster with time compared to that of S–VA–GF–1. Upon achieving a thermal steady state (at 110 s), the temperature of O–VA–GF–1 is slightly higher than that of S–VA–GF–1. The test setup depicted in Figure 4d is used to compare the transient heat‐transfer processes along the in‐plane direction of both complexes. The center of the sample is illuminated using a spot‐like lase‐light source, and the real‐time temperature evolution is recorded using an IR thermal imager. When the light source is alternately switched on and off, and its power increased in a gradient, O–VA–GF–1 invariably reaches the temperature inflection point at either P1 or P2 faster than S–VA–GF–1 (Figure 4e,f), indicating that it has excellent in‐plane heat‐transfer capability. Notably, the surface temperature of O–VA–GF–1 is lower than that of S–VA–GF–1. The higher through‐plane thermal conductivity of O–VA–GF–1 allowed the heat generated by the light source on the surface to be quickly transferred to the substrate (Al_2_O_3_) for heat dissipation. Furthermore, this result corroborates the aforementioned analysis of the heat transfer along the through‐plane direction (Figure 4a–c). It provides technical guidance for the heat dissipation application of point or surface heat sources. In addition, the temperature difference at P1 and P2 (ΔT) of O–VA–GF–1 and S–VA–GF–1 is plotted simultaneously as a function of the power percentage as shown in Figure 4g. The former difference has a smaller ΔT and is independent of the power percentage, indicating a faster horizontal heat‐transfer efficiency and stability. The aforementioned results conclusively reveal that the dense, interleaved, and hierarchical structure of the O–VA–GF plays a critical role in achieving its improved performance in terms of comprehensive heat–transfer.

### 2.3 Thermal Management Performance of VA–GF Composites

**Figure 5:**
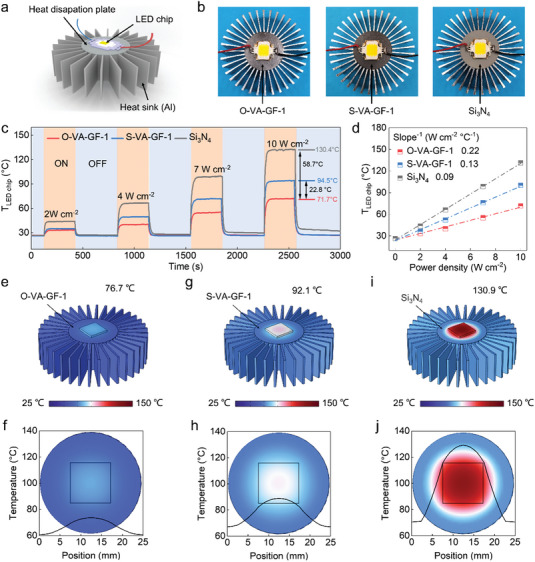
a) Scheme illustrating the use of a heat dissipation plate to connect the LED chip and “wind chimes” heat sink. b) Optical photographs displaying three cooling systems integrated with O–VA–GF–1, S–VA–GF–1, and Si_3_N_4_ as HDPs. c) Steady‐state center temperature evolution of the LED chips with respect to time at different power densities. d) Steady‐state center temperature of the LED chips as a function of power density. e) The temperature distribution of the cooling system of the integrated O–VA–GF–1 as the heat dissipation plate, with the corresponding temperature distributions illustrated in (f). g, h and i, j) The same cases for the S–VA–GF–1 and the Si_3_N_4_ as heat dissipation plate, respectively.

O–VA–GF–1 and S–VA–GF–1 show great potential as heat dissipation plates (HDPs) for efficiently cooling high‐power light‐emitting diode (LED) chips owing to their high *κ*_∥_ and *κ*_⊥_ values.^[^ ^9^, ^35^ ^]^ To test the thermal management performance of the HDPs, a verification system is designed to simulate the heat dissipation process of the electronic components (**Figure** **5**a). Before running the test, a silicon nitride (Si_3_N_4_, 70 W m^−1^ K^−1^) sheet, O–VA–GF–1, and S–VA–GF–1, featuring the same diameter of 25 mm, are first integrated as HDPs to connect an LED (rated power: 10 W) and a “wind chimes” heat sink. Meanwhile, thermally conductive silicone grease is used to secure the three parts to ensure good contact, further improving the interfaces of LED chip–heat dissipation plate–heat sink. Optical photographs of the experimental setup for the three systems are presented in Figure 5b. Subsequently, the temperature evolution of the LED chip's surface center with time is recorded using a calibrated thermocouple and an IR thermal imager. As indicated by the comparative results presented in Figure 5c, as the power density increases, the system integrated with O–VA–GF–1 is more advantageous compared to that integrated with S–VA–GF–1 and Si_3_N_4_ for cooling the high‐power LED. When the power density increases to 10 W cm^−2^, significant heat accumulates at the center of the LED chip in the system integrated with Si_3_N_4_, with the steady‐state temperature (130.4 °C) exceeding the node temperature (85 °C). For the system integrated by S–VA–GF–1, the steady‐state center temperature of the LED chip (94.5 °C) is slightly over 85 °C. Solely the LED chip in the system integrated with O–VA–GF–1 exhibits regular operation, and its temperature (71.7 °C) is 58.7 °C and 22.8 °C lower than that of the previous two systems, respectively. The corresponding IR thermal images of the three systems over time are consistent with the aforementioned analysis (Figure S17, Supporting Information). Notably, the operating temperature of an LED chip can significantly affect its working life, and every 10 °C drop in temperature increases its service life by 50%. Thus, such a significant reduction in the operating temperature is essential for extending the working life of the chip. Moreover, the equivalent heat‐transfer coefficients (equal to the reciprocal of the slope) of the three systems are calculated to be 0.09 W cm^−2^°C^−1^ (Si_3_N_4_), 0.13 W cm^−2^°C^−1^ (S–VA–GF–1) and 0.22 W cm^−2^°C^−1^ (O–VA–GF–1) (Figure 5d). This result indicates that the cooling efficiency of the system using O–VA–GF–1 as the heat dissipation plate is 144% higher than that of Si_3_N_4_. Additionally, the system using S–VA–GF–1 achieves a 44% enhancement in the cooling efficiency compared with the latter complex. Furthermore, the O–VA–GF–1 and S–VA–GF–1 also exhibit sufficient mechanical properties for use in HDPs. As illustrated in Figure S18a–d (Supporting Information), its tensile (24.8 MPa) and bending (39.4 MPa) strengths at 25 °C exceed those of S–VA–GF–1 (4.3 MPa and 29.8 MPa, respectively). And the O–VA–GF–1 also exhibits higher tensile and bending strengths from ‐30–150 °C compared to S–VA–GF–1, as shown in Figure S18e–h. The decrease in strength at ‐30 °C is related to an increase in stress concentration center due to the shrinkage of carbon crystals. However, because of the mismatch in the thermal expansion coefficient between the framework (VACNTs@SiC–GF) and the matrix (carbonized MP), thermal stresses should be induced in O–VA–GF–1 and S–VA–GF–1 after processing. The thermal stresses are gradually released with an increase in temperature to 150 °C, resulting in the increase of tensile and bending strengths.^[^ ^36^ ^]^ In addition, the O–VA–GF–1 and S–VA–GF–1 have no mass loss when the temperature is lower than 450 °C, but lose ≈2.45% and ≈16.05% at 600 °C, respectively. This result indicates that O–VA–GF–1 and S–VA–GF–1 exhibit thermal stability and oxidation resistance even at high temperatures in Figure S19 (Supporting Information).

To investigate the heat dissipation capability of the three systems, the FEA is performed under the same test conditions, with an input power density of 10 W cm^−2^ (the detailed simulation model and parameter settings are summarized in Figure S20 and Table S5, Supporting Information). Notably, the temperature distribution of the system is compared at different viewing angles. The resultant temperature distribution of the cooling system is presented in Figure 5e–j, and the simulated temperature distributions of the three systems (O–VA–GF–1, S–VA–GF–1, and Si_3_N_4_) are consistent with the experimental data, with steady‐state temperatures of 76.7, 92.1, and 130.9 °C, at the center of the LED chip, respectively (Figure 5e,g,i). The cooling system integrated with O–VA–GF–1 yields a more uniform temperature distribution along the in‐plane direction, as demonstrated in Figure 5f, compared with the systems integrated with S–VA–GF–1 (Figure 5h) and Si_3_N_4_ (Figure 5j). Moreover, the temperature at the interface generated by the heat dissipation plate and LED chip in the system using O–VA–GF–1 is significantly lower than that generated by the others (Figure S21, Supporting Information). This comparison illustrates that the excellent *κ*_⊥_ of O–VA–GF–1 plays an important role in the directional export of heat generated by the heater, whereas the ultrahigh *κ*_∥_ allows the accumulated heat to be extracted to the heat sink. In particular, the presence of large‐area GF on the O–VA–GF–1 surface enhances the contact area with the mating surfaces. This effectively prevents the formation of hot spots in the heat‐generation process of high‐power devices. Based on the superior heat‐transfer capability and good thermal stability of our all‐carbon‐based thermally conductive materials, the design of the VA–GF composites entails far‐reaching implications for high‐flux heat dissipation in cooling applications for electronics.

## 3 Conclusions

In summary, based on the force–thermal coupling concept, a hot‐pressing assembly strategy with pre‐fabricated stable micro–nano structure was proposed to fabricate compressed building blocks. This fabrication was achieved by incorporating an ordered interconnected multidimensional VACNTs@SiC–GF framework, which was prefilled with MP to form stable VANCTs@SiC–GF/MP, resulting in both high thermal conductivity and mechanical strength. The obtained hierarchical O–VA–GF formed an interleaved structure with inter–embedding of CNTs at the interface, thereby improving the support, maintaining the vertical orientation of the arrayed CNTs, and creating a dense fusion interface compared with S–VA–GF. The hierarchical structure of O–VA–GF enabled continuous multidimensional thermal pathways to enhance interfacial phonon‐transfer efficiency. Consequently, by modulating the assemble pressure, the resultant O–VA–GF and S–VA–GF exhibited the maximum *κ*_⊥_ of 41.7 and 10.3 W m^−1^ K^−1^, the corresponding *κ*_∥_ of 397.9 and 240.9 W m^−1^ K^−1^. Moreover, compared with previously reported carbon‐based HDMs, the hierarchical compressed building blocks attained excellent thermal conductivity and further optimized the anisotropic heat–transfer. Furthermore, the selection of O–VA–GF and S–VA–GF as heat dissipation plates for cooling high–power LED chips, respectively, yielded 144% and 44% improvements in equivalent heat‐transfer coefficients compared with that of conventional Si_3_N_4_, as verified through testing and FEA. The preferred O–VA–GF and S–VA–GF with sufficient tensile strength (24.8 and 4.3 MPa, respectively) and bending strength (39.4 and 29.8 MPa, respectively) could resist larger loadings, thereby fulfilling the mechanical requirements for using heat dissipation plates. In addition, we demonstrated that the formation of a strongly interactive supporting structure during the compression process could effectively weaken the strong orientation effect of the pressure, thereby improving the orientation of the materials perpendicular to the pressure direction to form hierarchical multidimensional thermally conductive compressed building blocks. The study findings reveal that VA–GF composites prove to be a promising candidate for the high‐flux heat dissipation of high‐power devices.

## 4 Experimental Section

### Materials

Tetraethyl orthosilicate (TEOS), polyvinylidene fluoride (PVDF), xylene, N–Methylpyrrolidone (NMP), KMnO_4_, concentrated H_2_SO_4_ (98%), H_2_O_2_, and HCl were obtained from Aladdin Co., Ltd. Ferrocene was purchased from Alfa Aesar Co. Ltd. Mesophase pitch (MP) was obtained from Wuhan Kexing Chemical Industry Co., Ltd. All the chemicals were used without further purification. Graphene oxide (GO) was synthesized by a modified Hummers method.^[^ ^37^ ^]^ Deionized water was prepared in the laboratory.

### Preparation of the SiO_2_–GF

Initially, 10 mg GO powder was dissolved in 10 mL deionized water by ultrasonication for 1 h at 25 °C. Subsequently, the dispersion was directly vacuum filtered through an anodic aluminum‐oxide‐based filter membrane with a pore size of 0.45 µm (Anodisc 47; Whatman®) to assemble a GO film (GOF). Subsequently, TEOS was spin coated onto the GOF surface. Finally, the film was sandwiched between two graphite plates and heated at 1000 °C for 1 h with 20 sccm of H_2_ and 200 sccm of Ar to obtain a SiO_2_–decorated graphene film (SiO_2_–GF).

### Preparation of VACNTs@SiO_2_–GF

VACNTs@SiO_2_–GF was synthesized using the floating catalytic chemical vapor deposition. For this process, 0.2 g Ferrocene (catalyst) was dissolved in 20 mL of xylene (carbon source) to prepare a precursor solution with a concentration of 0.01 g mL^−1^. The as‐prepared SiO_2_–GF was subsequently fixed to a quartz sheet and placed at the center of the quartz tube. Subsequently, the temperature of the tube furnace was increased to 860 °C at a rate of 10 °C min^−1^ with 150 sccm of H_2_ and 1000 sccm of Ar, while the precursor solution was injected into the quartz tube at a rate of 20 mL h^−1^ using a syringe pump, obtaining the VACNTs@SiO_2_–GF.

### Preparation of VACNTs@SiC–GF

The as‐prepared VACNTs@SiO_2_–GF was placed at the center of the quartz tube, and the temperature of the tube furnace was then increased to 1700 °C at a rate of 10 °C min^−1^ with 500 sccm of Ar for 30 min to obtain the VACNTs@SiC–GF.

### Preparation of VA–GF Composites

MP (5 g) and PVDF (50 mg) were dissolved in NMP (50 mL), followed by sonication for 1 h, to obtain a homogeneous MP–PVDF/NMP suspension. The VACNTs@SiC–GF was impregnated at 100 °C in a Teflon mold containing the suspension to obtain a VACNTs@SiC–GF/MP. Two VACNTs@SiC–GF/MP were placed into a graphite mold (denoted as VACNTs aligned to VACNTs or VACNTs aligned to graphene, respectively) and thereafter transferred to a vacuum hot‐pressing furnace. The furnace was initially heated to 300 °C whereas the pressure was set to 0.1, 1, and 10 MPa, and heating was continued to 2000 °C for 1 h. Finally, a series of corresponding O–VA–GF (O–VA–GF–0.1, O–VA–GF–1, and O–VA–GF–10) and S–VA–GF (S–VA–GF–0.1, S–VA–GF–1, and S–VA–GF–10) were obtained after the furnace naturally cooled to 25 °C.

### Structural Analysis and Characterization

The microstructures of the materials were observed using field–emission scanning electron microscopy (SEM; Sigma 300, ZEISS, Germany) and high–resolution transmission electron microscopy (TEM; Tecnai G2 F20, FEI, USA).

The crystal structure and surface chemical compositions were determined using X–ray diffraction analysis (XRD; D8 Advance, Bruker, Germany) with Cu K*α* radiation (*λ* = 1.54 Å) and X–ray photoelectron spectroscopy (XPS; K–Alpha^+^, Thermo Fisher, USA). Small–angle X–ray scattering (SAXS) experiments were performed using a 2D SAXS instrument (Xeuss 2.0, Xenocs, France), and the sample–to–detector distance was fixed at 2490 mm. A flat–panel detector (Pilatus 300 K) was employed to collect data with a wavelength of 1.5412 Å and resolution of 487×619 pixels. Hermans orientation function (*f*) of the representative crystalline lattice is defined as follows^[^ ^23^, ^24^ ^]^:

(5) $$f=\frac{3\langle cos^{2}\varphi \rangle -1}{2}$$

(6) $$cos^{2}\varphi =\frac{\int_{0}^{\frac{\pi }{2}}\;I\left(\varphi \right)\;cos^{2}\varphi \;sin\varphi \;d\varphi }{\int_{0}^{\frac{\pi }{2}}\;I\left(\varphi \right)\;sin\varphi \;d\varphi }$$

where *φ* denotes the angle between the normal direction of the crystalline lattice and the reference axis (equatorial direction).

### Performance Testing and Characterization—Thermal Conductivity

The thermal diffusivity (*α*) was measured using a laser flash apparatus (LFA 467, Netzsch, Germany), the specific heat capacity (*C*_p_) was measured using a differential scanning calorimeter (DSC) (Q20, TA Instruments, USA), and the density (*ρ*) was determined using the water displacement method. The thermal stability in an air environment was performed using the thermogravimetric analysis (TGA) (TGA55, TA instruments, USA).

Surface temperature evolution and thermal images were captured using an infrared thermal imager (Tix640, Fluke, USA).

### Performance Testing and Characterization—Mechanical Properties

The tensile performance was tested using an electronic universal testing machine (XQ–1C, Xin Xian, China) using samples 20 mm (length) ×5 mm (width) in size at a tensile rate of 5 mm min^−1^. Moreover, the bending performance was tested using samples 40 mm (length) ×10 mm (width) in size at a rate of 1 mm min^−1^.

## Conflict of Interest

The authors declare no conflict of interest.

## Supporting information

Supporting Information

## Data Availability

The data that support the findings of this study are available in the supplementary material of this article.
