# Supplementary material for: Tailoring Dense, Orientation–Tunable, and Interleavedly Structured Carbon‐Based Heat Dissipation Plates
Source: Adv Sci (Weinh). 2023 Jan 10;10(7):2205962. doi: 10.1002/advs.202205962 (PMC9982569; doi:10.1002/advs.202205962)
Supplement: Supplementary file 1 — Supporting Information [file ADVS-10-2205962-s001.pdf]

ADVANCED SCIENCE
Open Access

Supporting Information

for *Adv. Sci.*, DOI 10.1002/advs.202205962

Tailoring Dense, Orientation–Tunable, and Interleavedly Structured Carbon-Based Heat Dissipation Plates

Lianqiang Peng, Huitao Yu, Can Chen, Qingxia He, Heng Zhang, Fulai Zhao, Mengmeng Qin, Yiyu Feng and Wei Feng**

Supporting Information

Tailoring Dense, Orientation-tunable, and Interleavedly Structured Carbon-based Heat Dissipation Plates

Lianqiang Peng, Huitao Yu, Can Chen, Qingxia He, Heng Zhang, Fulai Zhao, Mengmeng Qin, Yiyu Feng, and Wei Feng**

Dr. L. Peng, Dr. H. Yu, Dr. C. Chen, Dr. Q. He, Dr H. Zhang, Dr. F. Zhao, Dr. M. Qin, Prof. Y. Feng, Prof. W. Feng
School of Materials Science and Engineering and Tianjin Key Laboratory of Composite and Functional Materials, Tianjin University, Tianjin 300350, P. R. China

Prof. Y. Feng
Key Laboratory of Materials Processing and Mold, Ministry of Education, Zhengzhou University, Zhengzhou 450002, P. R. China

*E-mail: weifeng@tju.edu.cn (W. Feng), fengyiyu@tju.edu.cn (Y. Feng).

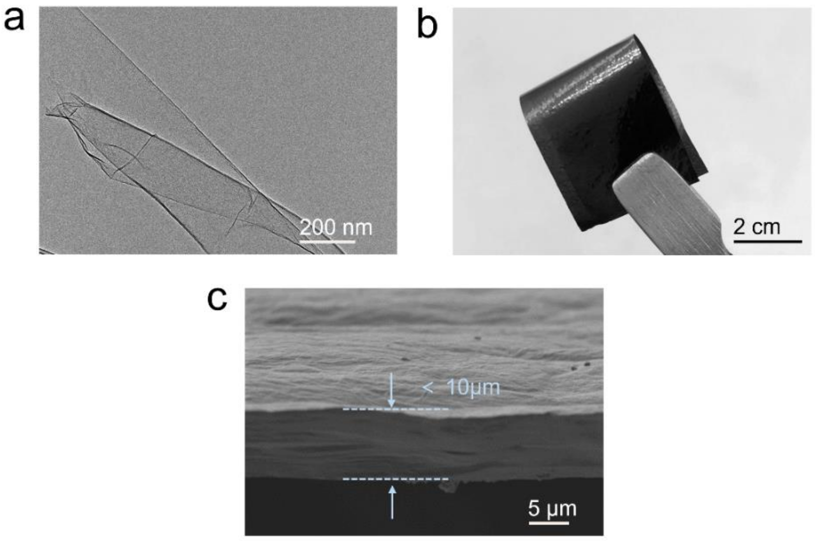

Figure S1. (a) TEM image of graphene oxide nanoflakes (b) Optical photograph and (c) SEM image of graphene oxide film.

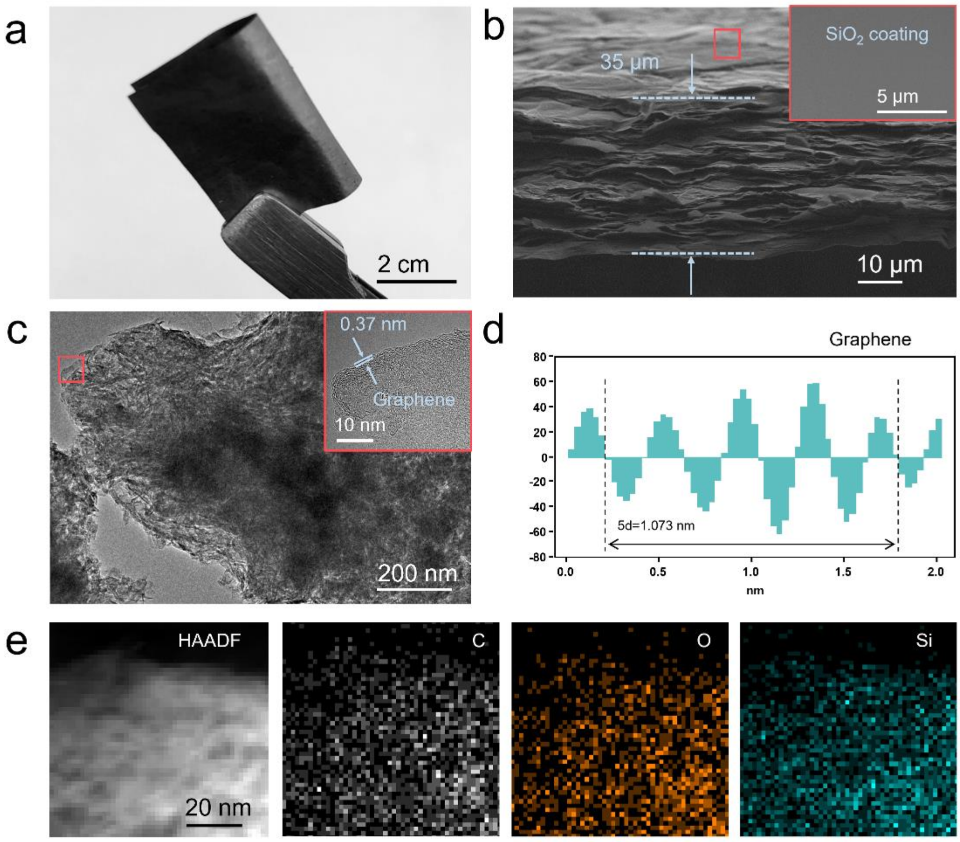

Figure S2. (a) Optical photograph, (b) SEM image (The inset shows the HRSEM image of the red boxed area), (c) TEM image (The inset shows the HRTEM image of the red boxed area) of SiO_2–GF. (d) The crystal plane spacing of graphene and (e) EDS mapping of the red boxed area in Figure S2c.

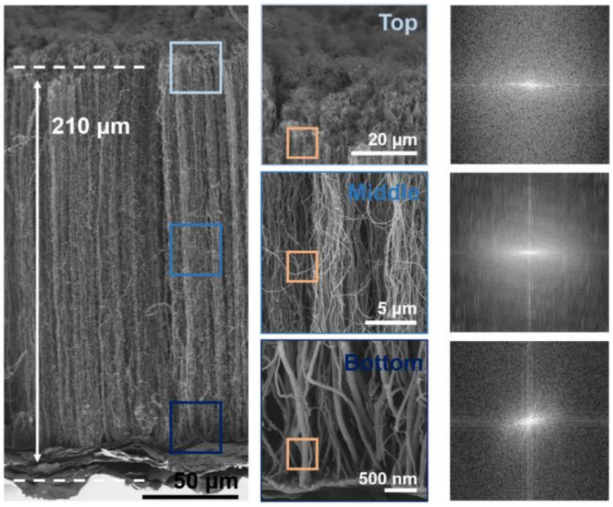

Figure S3. SEM images of VACNTs@SiC–GF show that the orientation of the VACNTs always remains vertically oriented from the top transition to the bottom. After FFT processing of the SEM images, the corresponding diffraction patterns are obtained of each position, which are always linear.

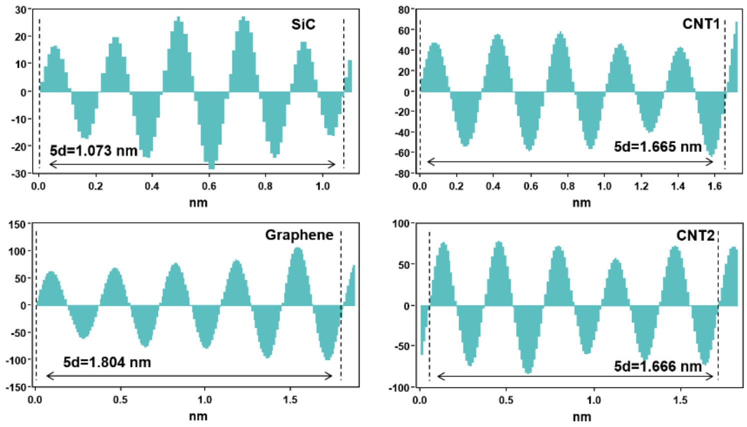

Figure S4. The crystal plane spacing of SiC, CNT1, graphene and CNT2 belonging to VANCTs@SiC–GF.

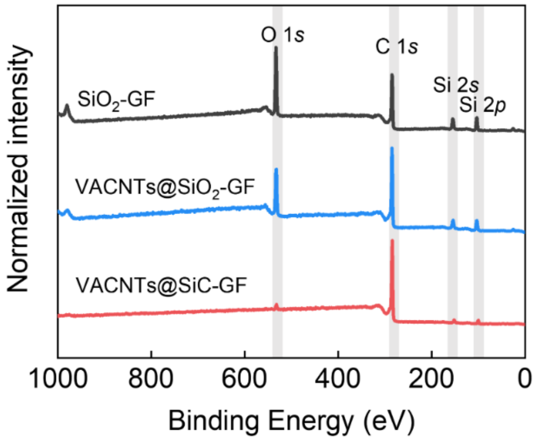

Figure S5. The XPS survry of the SiO_2–GF, VACNTs@SiO_2–GF and VACNTs@SiC–GF.

First-principle calculations

We have employed the Vienna Ab Initio Package (VASP) [S1]to perform all the density functional theory (DFT) calculations within the generalized gradient approximation (GGA) using the PBE [S2] formulation. We have chosen the projected augmented wave (PAW) potentials [S3] to describe the ionic cores and take valence electrons into account using a plane wave basis set with a kinetic energy cutoff of 400 eV. Partial occupancies of the Kohn−Sham orbitals were allowed using the Gaussian smearing method and a width of 0.05 eV. The electronic energy was considered self-consistent when the energy change was smaller than 10^{-5} eV. A geometry optimization was considered convergent when the force change was smaller than 0.02 eV/Å. Grimme's DFT-D3 methodology [S4] was used to describe the dispersion interactions.

The equilibrium lattice constant of hexagonal graphene unit cell separated by a vacuum layer in the depth of 15 Å was optimized, when using a 15×15×15 Monkhorst-Pack k-point grid for Brillouin zone sampling, to be a=2.468 Å. The equilibrium lattice constant of cubic β-SiO_2 unit cell was optimized, when using a 4×4×4 Monkhorst-Pack k-point grid for Brillouin zone sampling, to be a=7.440 Å. The equilibrium lattice constant of cubic β-SiC unit cell was optimized, when using a 11×11×11 Monkhorst-Pack k-point grid for Brillouin zone sampling, to be a=4.368 Å. atoms were allowed to relax.

The CNT/SiO_2(001)/graphene heterojunction (VACNTs@ SiO_2–GF) was built; the CNT part comprises of 48 C atoms. the SiO_2(001) part has a p (2×3) periodicity in the X and Y

directions and one stoichiometric layer in the Z direction; the graphene part has a *p* ($6\times5\cdot3^{0.5}$) periodicity in the X and Y directions and one monolayer in the Z direction. During structural optimizations, the gamma point in the Brillouin zone was used for k-point sampling, and all

The CNT/SiC (100)/graphene heterojunction (VACNTs@SiC–GF) was built; the CNT part comprises of 48 C atoms. the SiC (100) part has a *p* (4×3) periodicity in the X and Y directions and one stoichiometric layer in the Z direction; the graphene part has a ($7\times3\cdot3^{0.5}$) periodicity in the X and Y directions and one monolayer in the Z direction. During structural optimizations, the gamma point in the Brillouin zone was used for k-point sampling, and all atoms were allowed to relax.

The binding energy (E_b) was calculated using the following equation:[S5]

$$E_b = E_{A/surf} - E_{surf} - E_{A(g)}$$

where $E_{A/surf}$ is total energy of VACNTs@SiO_2–GF and VACNTs@SiC–GF systems, E_{surf} is the energy of SiO_2-GF or SiC-GF, and $E_{A(g)}$ is the energy of VACNTs. The energy of A molecule in a cubic periodic box with a side length of 20 Å and a 1×1×1 Monkhorst-Pack k-point grid for Brillouin zone sampling, respectively. As shown in **Table S2**, the calculated binding energy for VACNTs@SiO_2–GF and VACNTs@SiC–GF systems were given. The corresponding optimized location of VACNTs@SiO_2–GF and VACNTs@SiC–GF were shown in **Figure S5**.

In addition, we also calculated the charge density difference $\Delta\rho$ of VACNTs@SiO_2–GF and VACNTs@SiC–GF systems using the following formula:[S6]

$$\Delta\rho = \rho_{A/surf} - \rho_{surf} - \rho_{A(g)}$$

where $\rho_{A/surf}$ was the charge density distribution of VACNTs@SiO_2–GF or VACNTs@SiC–GF

systems, ρ_{surf} was the charge density distribution of SiO_2–GF or SiC–GF, and $\rho_{A(g)}$ was the charge density distribution of VANCTs. The corresponding charge density distribution of VACNTs@SiO_2–GF and VACNTs@SiC–GF were given systems in **Figure S6** and **S7**.

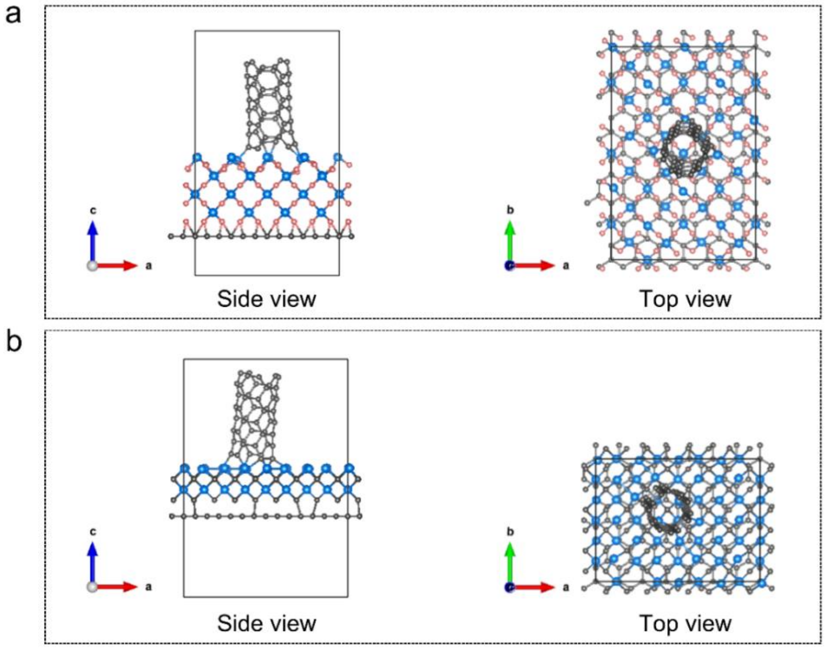

Figure S6. The optimized location of (a) VACNTs@SiO_2–GF and (b) VACNTs@SiO_2–GF systems based on DFT simulations.

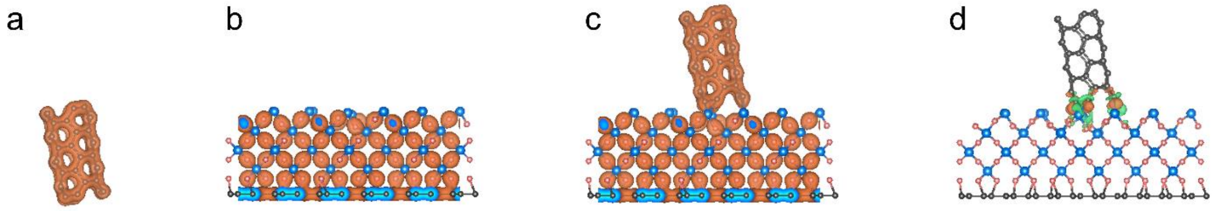

Figure S7. Charge density distribution of (a) VANCTs (b) SiO_2–GF, and (c) VACNTs@SiO_2–GF constructed by DFT calculations. The isosurface contours correspond to 0.1 e Å^{-3} (d) Charge density difference of VACNTs@SiO_2–GF. The isosurface contours correspond to 0.01 e Å^{-3}.

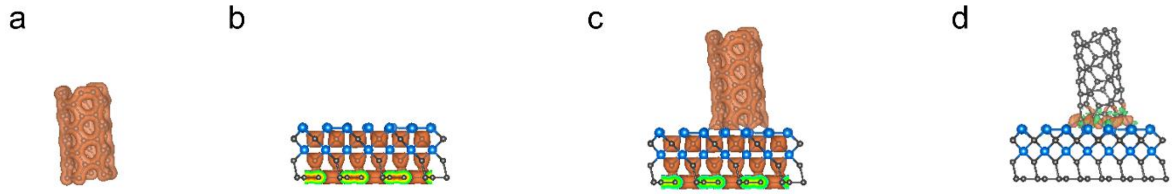

Figure S8. Charge density distribution of (a) VANCTs (b) SiC–GF, and (c) VACNTs@SiC–GF constructed by DFT calculations. The isosurface contours correspond to 0.1 e Å^{-3} (d) Charge density difference of VACNTs@SiC–GF. The isosurface contours correspond to 0.01 e Å^{-3}.

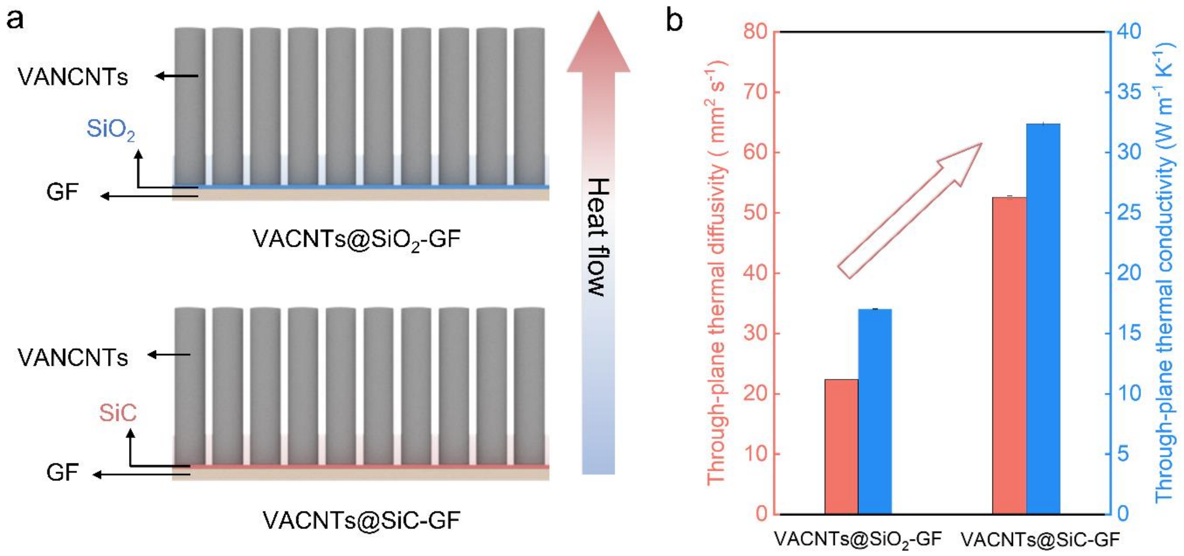

Figure S9. (a) Schematic illustration of the heat-transfer mechanism of VACNTs@SiO_2–GF and VACNTs@SiC–GF along the through-plane. (b) The through-plane thermal diffusivities and thermal conductivities of the VACNTs@SiO_2–GF and VACNTs@SiC–GF.

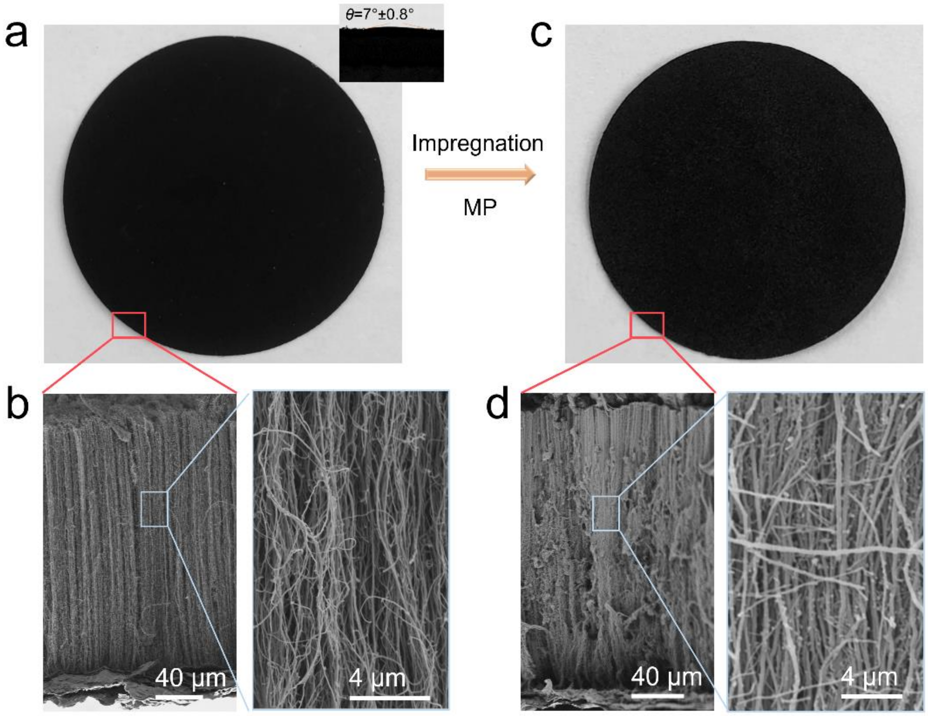

Figure S10. (a) Optical photograph (Inset shows sessile drop of MP–PVDF/NMP on the top surface of the VACNTs@SiC–GF. The measured contact angle is 7° ± 0.8°.) and (b) the SEM image of VACNTs@SiC–GF. (c, d) The same case for the VACNTs@SiC–GF /MP, respectively.

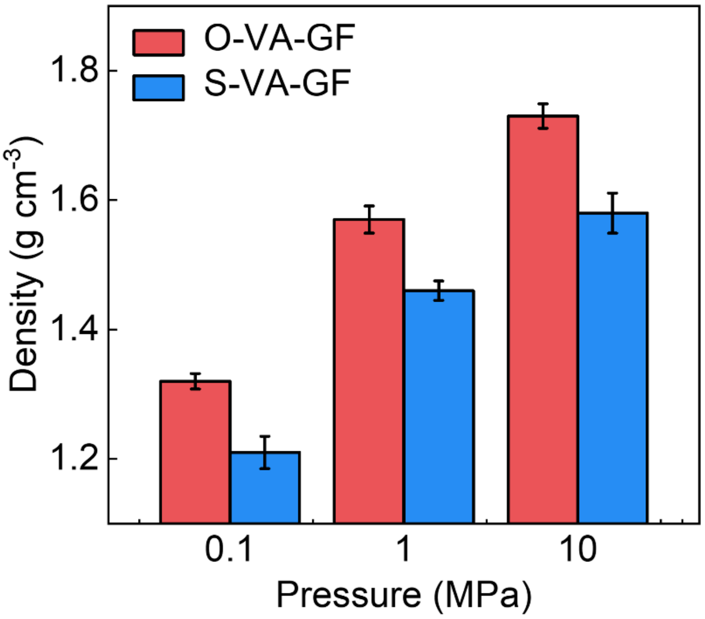

Figure S11. The density of O–VA–GF and S–VA–GF as a function of pressure.

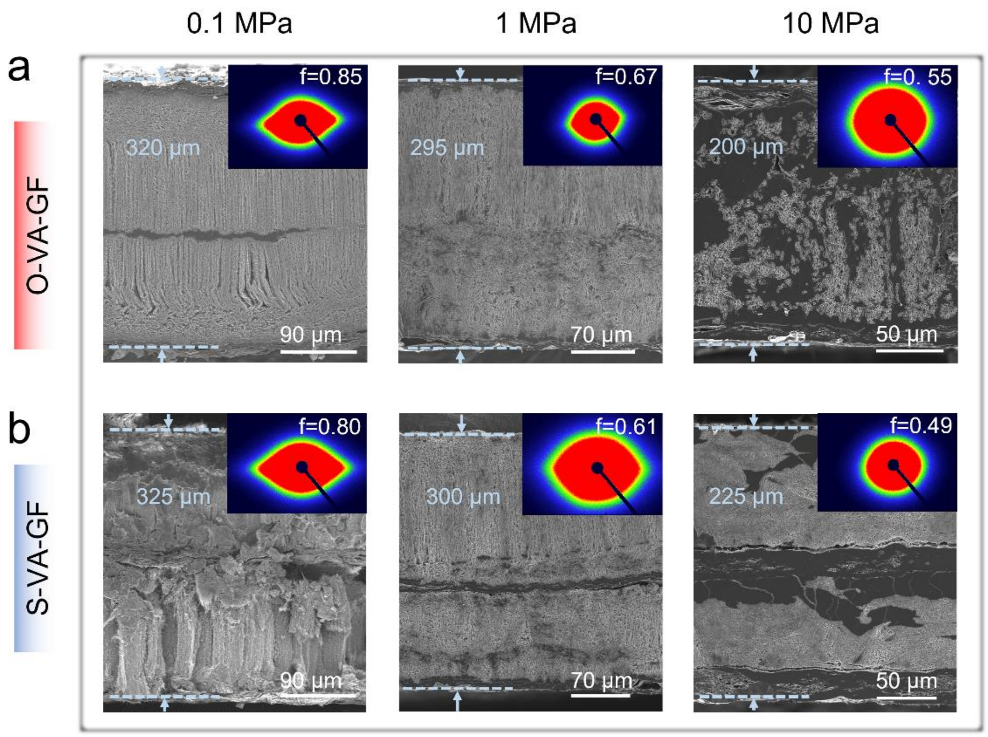

Figure S12. SEM images and 2D SAXS patterns of (a) O–VA–GF and (b) S–VA–GF.

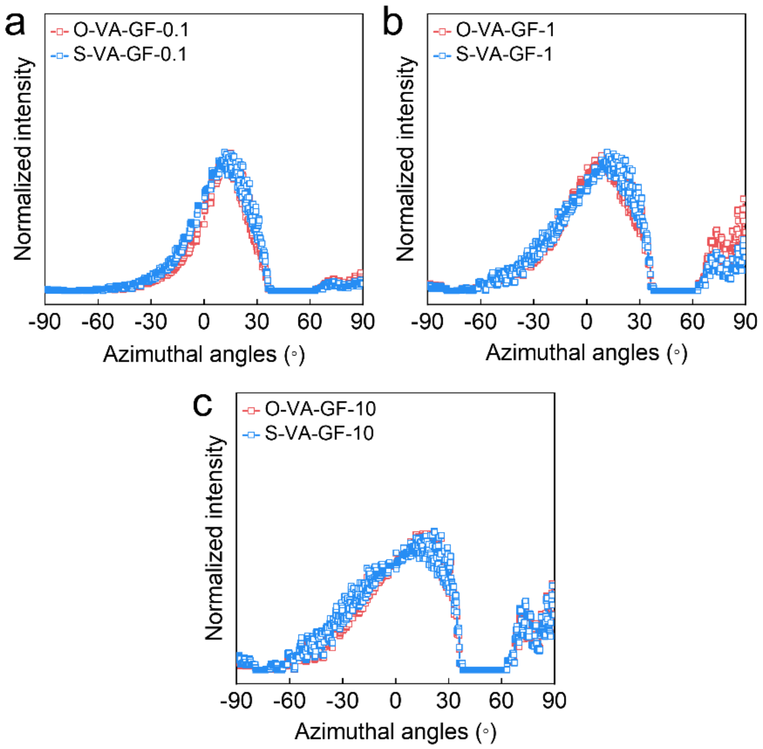

Figure S13. The azimuthal angle distributions for (a) O–VA–GF–0.1/S–VA–GF–0.1, (b) O–VA–GF–1/S–VA–GF–1, and (c) O–VA–GF–10/S–VA–GF–10.

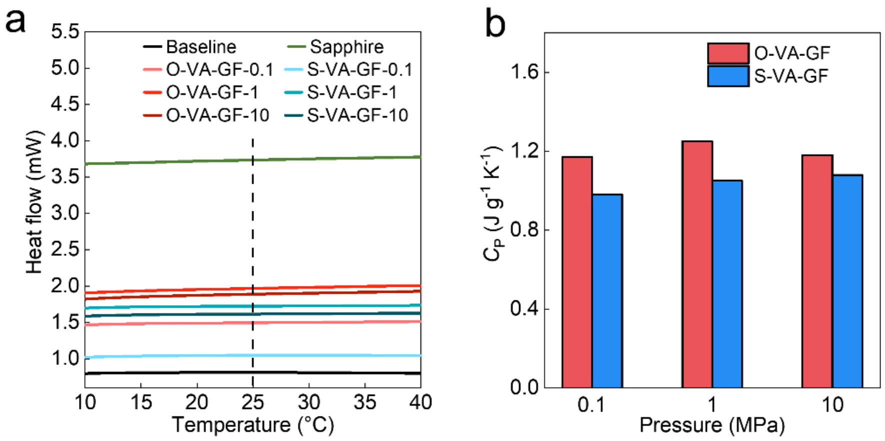

Figure S14. (a) DSC curves of O–VA–GF (O–VA–GF–0.1, O–VA–GF–1 and O–VA–GF–10), S–VA–GF (S–VA–GF–0.1, S–VA–GF–1 and S–VA–GF–10), empty sample and sapphire. (b) Specific heat capacity (C_p) of O–VA–GF and S–VA–GF calculated based on the following equation as a function of pressure.

$$C_p = \frac{\Delta_Q \times m_{Sapphire} \times C_{p\ Sapphire}}{\Delta_{Q\ Sapphire} \times m}$$

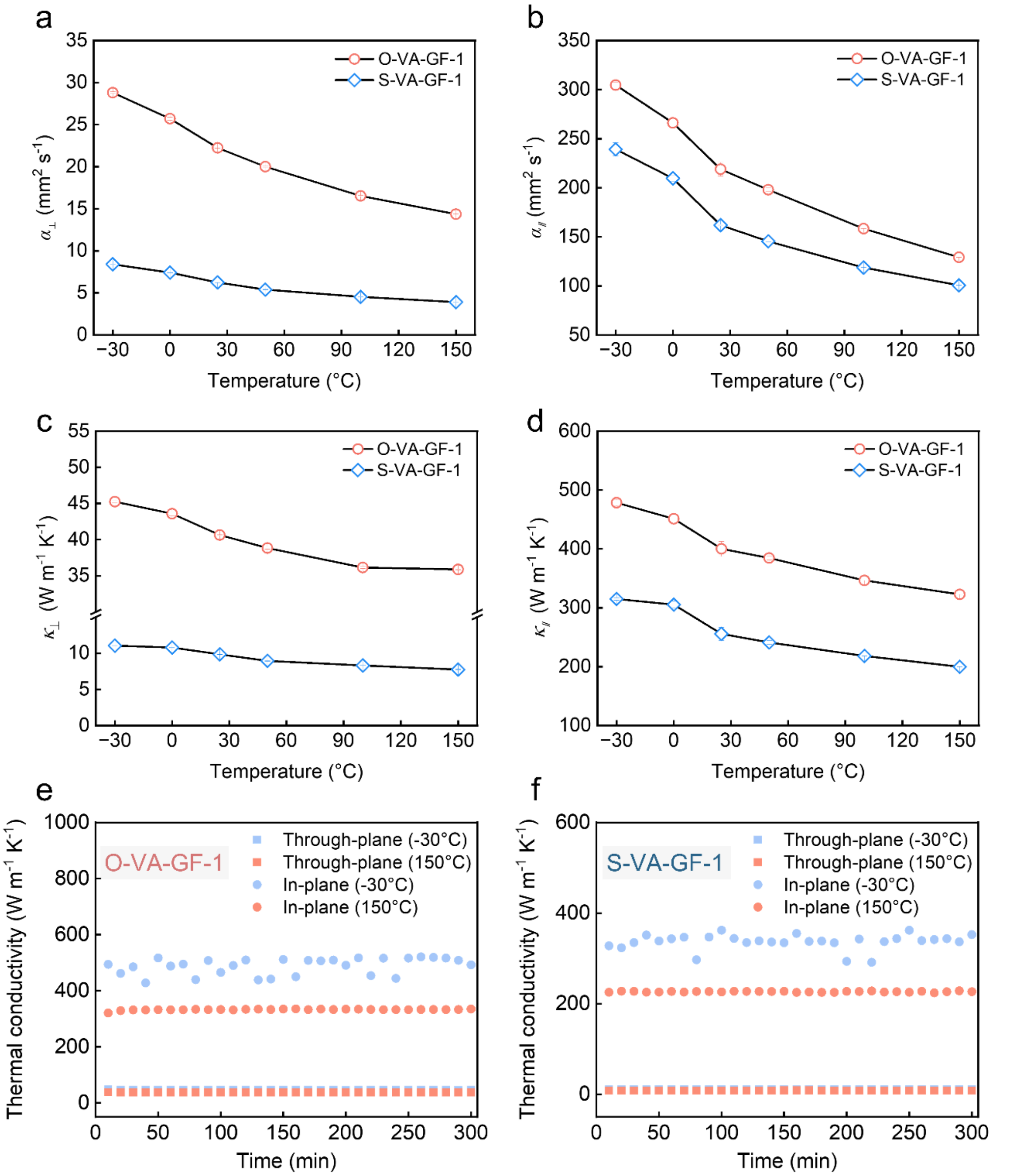

Figure S15. (a)$\alpha_\perp$, (b), $\alpha_{//}$ (c) $\kappa_\perp$ as well as (d) $\kappa_{//}$of the O–VA–GF–1 and S–VA–GF–1 as a function of environmental temperature. The thermal conductivities of (e) O–VA–GF–1 and (f) S–VA–GF–1 *versus* time at different temperatures.

Finite element analysis

Combining the experimental results shown in **Figure 3d** and the strain characteristics of VACNTs, the transient thermal response of the O–VA–GF (O–VA–GF–0.1, O–VA–GF–1 and O–VA–GF–10) and S–VA–GF (S–VA–GF–0.1, S–VA–GF–1 and S–VA–GF–10) during heating were simulated using Comsol 6.0 software.[S7] The corresponding simulation models were shown in **Figure S16**. In order to simplify the Comsol model, we replaced the GF, carbon nanotubes within the O–VA–GF and S–VA–GF with rectangles and cylinders of different curvature and orientation, as well as set the thermal conductivity of the GF and carbon nanotubes to 500 and 1000 W m^{-1} K^{-1}, respectively.[S8] And the interstitial regions between the carbon nanotubes all referred to the carbonized MP, with the thermal conductivity of 10 W m^{-1} K^{-1}.

The computational initial conditions of the six models were set to 25 °C, and adiabatic boundary conditions were specified at the starting state. Then, six constant heat sources with a temperature of 100 °C (schematically illustrated as the red line) were applied at the bottom of the O–VA–GF and S–VA–GF models, respectively, leading to the formation of one-dimensional heat conduction. When the transient analysis began, we set three groups of temperature probes located on the same positions of the six modules to measure the transient thermal response curves.

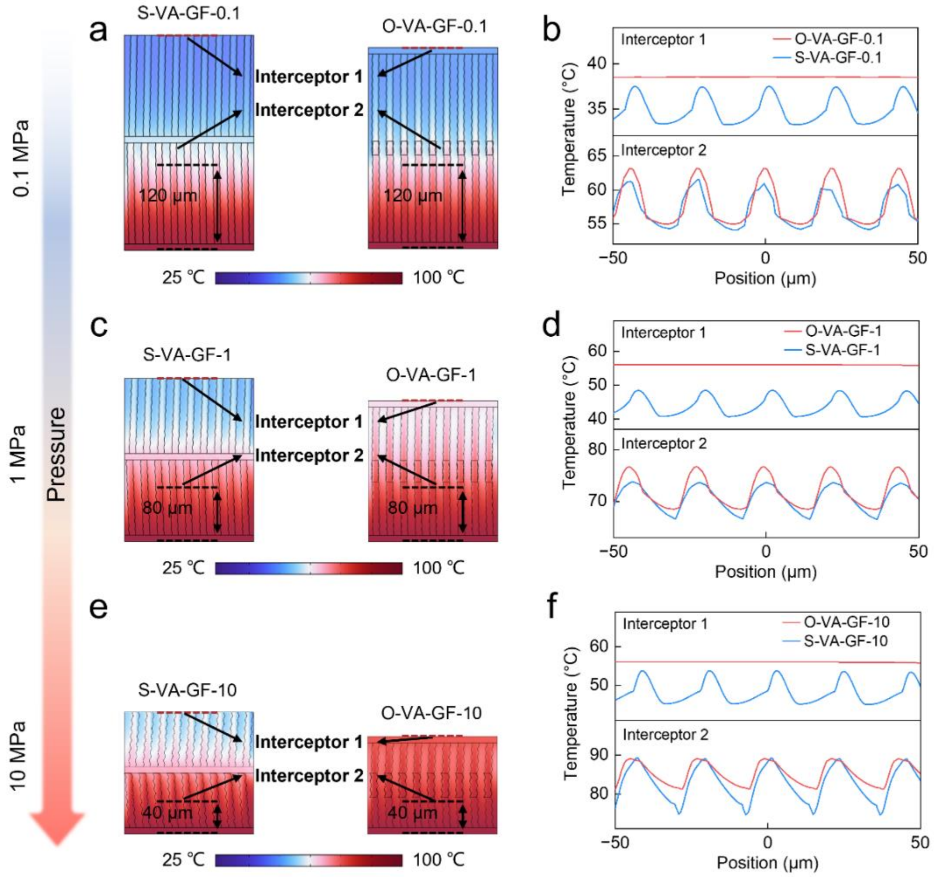

Figure S16. Simulated transient-temperature distribution for (a) the O–VA–GF–0.1/S–VA–GF–0.1, (c) O–VA–GF–1/S–VA–GF–1 and (e) O–VA–GF–10/S–VA–GF–10 modules. The temperature evolution of the measured interceptors versus the heating up time for (b) the O–VA–GF–0.1/S–VA–GF–0.1, (d) O–VA–GF–1/S–VA–GF–1 and (f) O–VA–GF–10/S–VA–GF–10 modules.

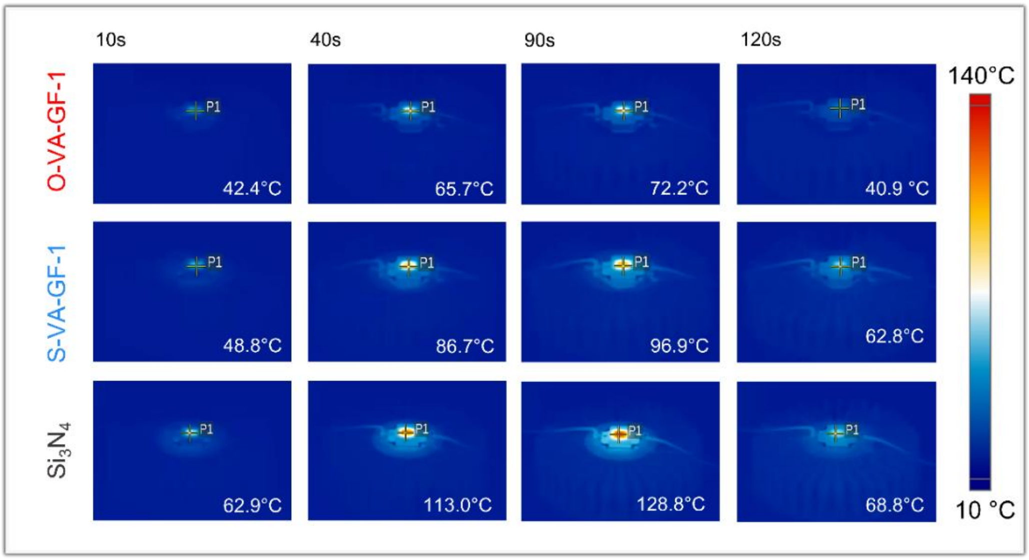

Figure S17. Comparative IR images when O–VA–GF–1, S–VA–GF–1 and Si_3N_4 were used as

HDPs, respectively.

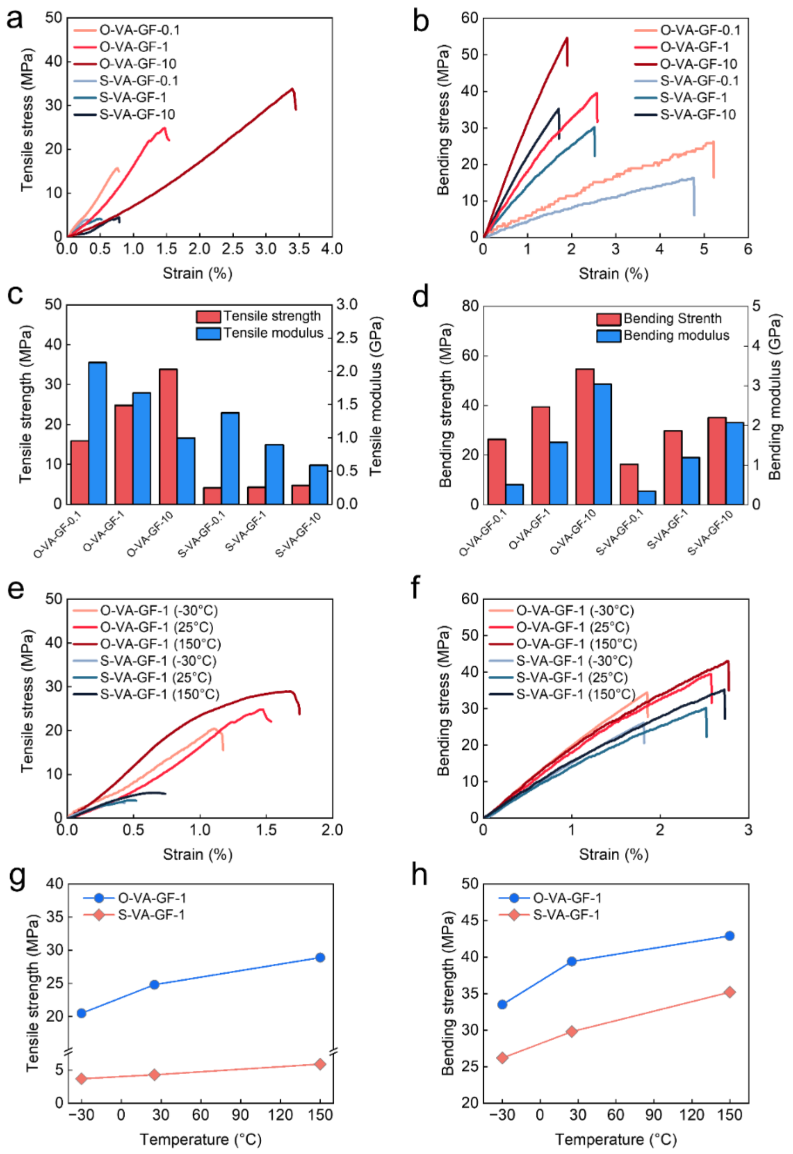

Figure S18. (a) Tensile stress-strain curves, (b) bending stress-strain curves, (c) tensile strength/modulus, and (d) bending strength/modulus of various samples. (e) Tensile stress-strain curves, (f) bending stress-strain curves, (g) tensile strength, and (h) bending strength of the O–VA–GF–1 and S–VA–GF–1 at various temperatures.

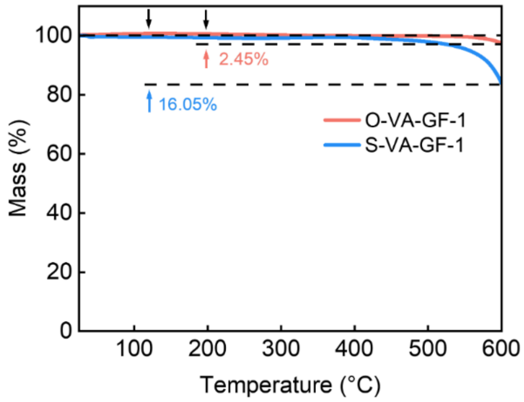

Figure S19. The TG thermograms of the O–VA–GF–1 and S–VA–GF–1.

Finite element analysis

To understand the role of the heat dissipation plates (HDPs), we used Comsol 6.0 software to simulate the heat transfer process of the cooling system.[S8b, S 9] The model implementation was shown in **Figure S20**, in which the power density of the heater (LED chip) was set to 10 W cm^{-2} and the background temperature of the whole system was set to 25 °C. The detailed parameters of the heater, heat sink, and the the three heat dissipation plates (O–VA–GF–1, S–VA–GF–1, Si_3N_4) were listed in **Table S4**. **Figure S21** presented the simulated cross-sectional temperature distribution of the cooling system.

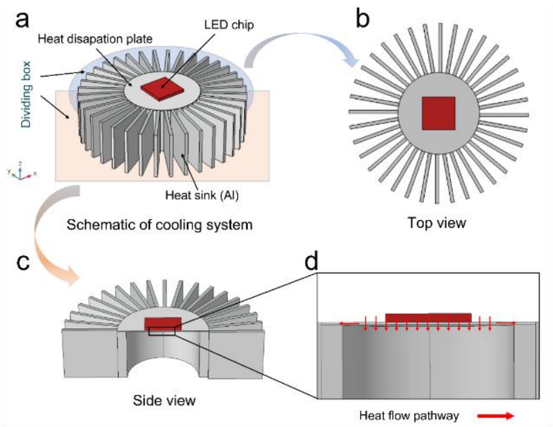

Figure S20. (a) Comsol system model for evaluating the thermal management performance of the HDPs. (b) The top view and (c) side view of the cooling system. (d) The integration of the dissipation plate on a "wind chimes" heat sink for cooling the LED chip with the heat flow pathway.

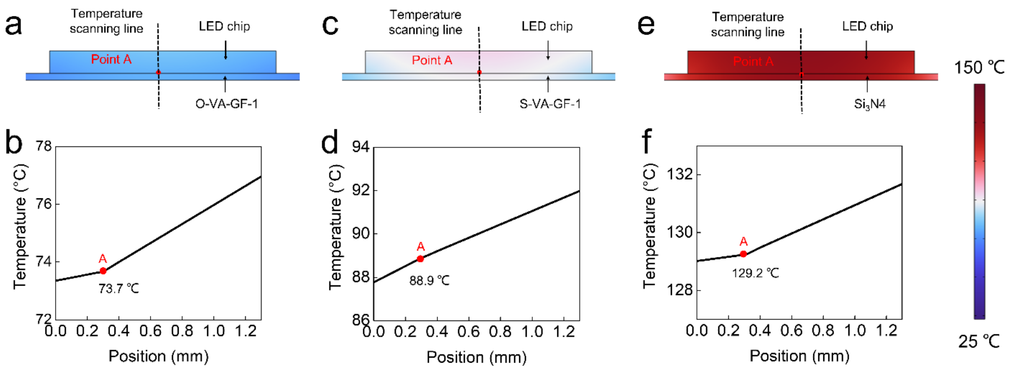

Figure S21. (a, c, e) Temperature distribution across the cross-section of the simulated system with HDPs (O–VA–GF–1, S–VA–GF–1 and Si_3N_4) and (b, d, f) the corresponding detailed temperature profiles.

Table S1. The XPS compositional analysis of our samples.

Sample	Elements	Binding energy (eV)	FWHM (eV)	Ratio (%)	Groups
SiO_2–GF	C 1*s*	284..50	1.08	64.31	C-C
		285.20	1.34	23.03	C=C
		286.30	2.23	12.66	C-O
	Si 2*p*	101.80	1.2	3.54	Si-O-C
		103.50	2.13	96.46	Si-O-Si
VACNTs@ SiO_2–GF	C 1*s*	284.50	1.02	68.69	C-C
		285.20	1.36	17.53	C=C
		286.10	1.82	11.10	C-O
		283.20	1.00	2.68	C-Si-O
	Si 2*p*	100.30	2.30	15.28	Si- C
		101.70	2.03	76.29	Si-O-C
		102.90	1.84	8.43	Si-O-Si
VACNTs@ SiC–GF	C 1*s*	284.50	0.98	70.74	C-C
		285.20	1.29	16.14	C=C
		286.00	2.05	10.41	C-O
		282.90	1.02	2.70	C-Si
	Si 2*p*	100.55	1.46	80.81	Si- C
		101.70	1.86	19.19	Si-O-C

Table S2. Binding energy of VACNTs@SiO_2–GF and VACNTs@SiC–GF system.

		E_total (eV)	Binding energy (eV)
System 1	SiO_2–GF	-2106.715	n/a
	VACNTs	-400.55	n/a
	VACNTs@SiO_2–GF	-2511.351	-4.09
System 2	SiC–GF	-1355.842	n/a
	VACNTs	-400.55	n/a
	VACNTs@SiC–GF	-1772.551	-16.16

Table S3. Comparison of in-plane and through-plane thermal conductivity of carbon-based thermally conductive composites.

Carbon-based thermally conductive composites	In-plane thermal conductivity (W m^{-1} K^{-1})	Through-plane thermal conductivity (W m^{-1} K^{-1})	Anisotropic ratio	Ref.
Carbon anoring/graphene hybrid Paper	946	2.1	450.5	S10
	901	3.8	237.1	
	896	5.8	154.5	
Graphene nanoplatelet paper	313	3.7	84.6	S11
	130	4.2	31.0	
	270	2.8	96.4	
	230	2.7	85.2	
	250	1.1	227.3	
Aligned single-wall carbon nanotube film	43	0.085	505.9	S12
Reduced graphene oxide/Multi walled carbon nanotube hybrid composite films	836.8	0.058	14427.6	S13
	804.24	0.061	13184.3	
	469.64	0.059	7960.0	
	414.61	0.089	4658.5	
Graphene/carbon nanotubes thick film	41.89	0.32	130.9	S14
	323.64	3.35	96.6	
	933.37	6.27	148.9	
	1021.26	1.16	880.4	
Graphene/silicon carbide nanorods film	123.5	10.9	11.3	S15
Natural graphite flakes/carbon nanotubes block	458.6	19.4	23.6	S16
	477.1	28.5	16.7	
Graphite/carbon nanotubes block	211.5	24.3	8.7	S17

Graphene-coated vertically aligned carbon nanotubes/graphite block	182.6	32.96	5.5	S18
O–VA–GF	490.9	17.7	27.7	This work
	397.9	41.7	9.5	
	290.3	36.5	8.0	
S–VA–GF	328.2	3.6	91.2	This work
	240.9	10.3	23.4	
	149.5	10.2	14.7	

Table S4. The C_P of the O–VA–GF–1 and S–VA–GF–1 at various temperatures.

Temperature (°C)	C_P of O–VA–GF–1 (J g^{-1} K^{-1})	C_P of O–VA–GF–1 (J g^{-1} K^{-1})
-30	1.00	0.90
0	1.08	1.00
25	1.17	1.08
50	1.24	1.20
100	1.39	1.26
150	1.59	1.36

Table S5. The detailed parameters of the components in the simulated system.

	Size (mm^3)	Materials	K (W m^{-1} K^{-1})	Cp (J g^{-1} K^{-1})
LED chip	10 × 10× 1	Alumina	27	0.91
Heat sink	Wind chimes	Aluminum	237	0.88
O–VA–GF–1	25 × 25× 0.3	—	397.9 (X-Y) / 41.7(Z)	1.17
S–VA–GF–1	25 × 25× 0.3	—	240.9 (X-Y) / 10.3 (Z)	1.08
Si_3N_4	25 × 25× 0.3	—	70	0.71

References

[S1]a)G. Kresse, J. Furthmüller, *Comput. Mater. Sci.* **1996**, *6*, 15; b)G. Kresse, J. Furthmüller, *Phys. Rev. B* **1996**, *54*, 11169.
[S2]J. P. Perdew, K. Burke, M. Ernzerhof, *Phys. Rev. Lett.* **1996**, *77*, 3865.
[S3]a)G. Kresse, D. Joubert, *Phys. Rev. B* **1999**, *59*, 1758; b)P. E. Blöchl, *Phys. Rev. B* **1994**, *50*, 17953.
[S4]S. Grimme, J. Antony, S. Ehrlich, H. Krieg, *J. Phys. Chem.* **2010**, *132*, 154104.
[S5]S. K. Jha, M. Roth, G. Todde, J. P. Buchanan, R. D. Moser, M. K. Shukla, G. Subramanian, *J. Phys. Chem. C* **2018**, *122*, 1288.
[S6]K. B. Wiberg, C. M. Hadad, T. J. LePage, C. M. Breneman, M. J. Frisch, *J. Phys. Chem.* **1992**, *96*, 671.
[S7]A. Van Schijndel, *Build. Simul.* **2009**, *2*, 143.
[S8]a)A. A. Balandin, *ACS nano* **2020**, *14*, 5170; b)A. Desai, S. Mahajan, G. Subbarayan, W. Jones, J. Geer, B. Sammakia, *J. Electron. Packag.* **2006**, *128*, 92.
[S9]a); b)J. Ying, X. Tan, L. Lv, X. Wang, J. Gao, Q. Yan, H. Ma, K. Nishimura, H. Li, J. Yu, *ACS nano* **2021**, *15*, 12922.
[S10]J. Zhang, G. Shi, C. Jiang, S. Ju, D. Jiang, *Small* **2015**, *11*, 6197.
[S11]a); b)H. Wu, L. T. Drzal, *Carbon* **2012**, 50, 1135.
[S12]S. Yamaguchi, I. Tsunekawa, N. Komatsu, W. Gao, T. Shiga, T. Kodama, J. Kono, J. Shiomi, *Appl. Phys. Lett.* **2019**, *115*, 223104.
[S13]T.-W. Pan, W.-S. Kuo, N.-H. Tai, *Compos. Sci. Technol.* **2017**, *151*, 44.
[S14]H. Jia, Q.-Q. Kong, X. Yang, L.-J. Xie, G.-H. Sun, L.-L. Liang, J.-P. Chen, D. Liu, Q.-G. Guo, C.-M. Chen, *Carbon* **2021**, *171*, 329.
[S15]W. Dai, L. Lv, J. Lu, H. Hou, Q. Yan, F. E. Alam, Y. Li, X. Zeng, J. Yu, Q. Wei, X. Xu, J. Wu, N. Jiang, S. Du, R. Sun, J. Xu, C. P. Wong, C. T. Lin, *ACS Nano* **2019**, *13*, 1547.
[S16]Y. Zhao, J. Shi, H. Wang, Z. Tao, Z. Liu, Q. Guo, L. Liu, *Carbon* **2013**, *51*, 427.
[S17]W. Feng, M. Qin, P. Lv, J. Li, Y. Feng, *Carbon* **2014**, *77*, 1054.
[S18]F. Lv, M. Qin, F. Zhang, H. Yu, L. Gao, P. Lv, W. Wei, Y. Feng, W. Feng, *Carbon* **2019**, *149*, 281.
